# The value of machine learning approaches in the diagnosis of early gastric cancer: a systematic review and meta-analysis

**DOI:** 10.1186/s12957-024-03321-9

**Published:** 2024-02-01

**Authors:** Yiheng Shi, Haohan Fan, Li Li, Yaqi Hou, Feifei Qian, Mengting Zhuang, Bei Miao, Sujuan Fei

**Affiliations:** 1 Department of Gastroenterology, The Affiliated Hospital of Xuzhou Medical University, 99 West Huaihai Road, Jiangsu Province, 221002 Xuzhou China; 2 First Clinical Medical College, Xuzhou Medical University, Jiangsu Province, 221002 Xuzhou China; 3 Institute of Digestive Diseases, Xuzhou Medical University, 84 West Huaihai Road, Jiangsu Province, 221002 Xuzhou China; 4 Key Laboratory of Gastrointestinal Endoscopy, Xuzhou Medical University, Jiangsu Province, 221002 Xuzhou China; 5 College of Nursing, Yangzhou University, Yangzhou, 225009 China

**Keywords:** Machine learning, Gastric cancer, Artificial intelligence, Endoscopy, Neural networks

## Abstract

**Background:**

The application of machine learning (ML) for identifying early gastric cancer (EGC) has drawn increasing attention. However, there lacks evidence-based support for its specific diagnostic performance. Hence, this systematic review and meta-analysis was implemented to assess the performance of image-based ML in EGC diagnosis.

**Methods:**

We performed a comprehensive electronic search in PubMed, Embase, Cochrane Library, and Web of Science up to September 25, 2022. QUADAS-2 was selected to judge the risk of bias of included articles. We did the meta-analysis using a bivariant mixed-effect model. Sensitivity analysis and heterogeneity test were performed.

**Results:**

Twenty-one articles were enrolled. The sensitivity (SEN), specificity (SPE), and SROC of ML-based models were 0.91 (95% CI: 0.87–0.94), 0.85 (95% CI: 0.81–0.89), and 0.94 (95% CI: 0.39–1.00) in the training set and 0.90 (95% CI: 0.86–0.93), 0.90 (95% CI: 0.86–0.92), and 0.96 (95% CI: 0.19–1.00) in the validation set. The SEN, SPE, and SROC of EGC diagnosis by non-specialist clinicians were 0.64 (95% CI: 0.56–0.71), 0.84 (95% CI: 0.77–0.89), and 0.80 (95% CI: 0.29–0.97), and those by specialist clinicians were 0.80 (95% CI: 0.74–0.85), 0.88 (95% CI: 0.85–0.91), and 0.91 (95% CI: 0.37–0.99). With the assistance of ML models, the SEN of non-specialist physicians in the diagnosis of EGC was significantly improved (0.76 vs 0.64).

**Conclusion:**

ML-based diagnostic models have greater performance in the identification of EGC. The diagnostic accuracy of non-specialist clinicians can be improved to the level of the specialists with the assistance of ML models. The results suggest that ML models can better assist less experienced clinicians in diagnosing EGC under endoscopy and have broad clinical application value.

**Supplementary Information:**

The online version contains supplementary material available at 10.1186/s12957-024-03321-9.

## Background

Gastric cancer (GC) is among the most prevailing gastrointestinal malignancies. Global Cancer Statistics [1] indicated that in 2020, there were 1,089,103 newly diagnosed GC patients and 768,793 GC-caused deaths, with morbidity ranking 5th and mortality ranking 4th among all types of cancer. This makes it a great hazard to public health worldwide [2]. The popularity of endoscopic screening, the improvements in comprehensive treatment strategies and surgical modalities, and the effective treatment of *Helicobacter pylori* (HP) infection in recent years have reduced the morbidity of GC, while the patients still have poor 5-year survival [3]. The median survival differs between the patients at an early stage and those at an advanced stage. Endoscopic therapy is recommended for GC patients staged T1 by the AJCC-TNM system. The 5-year survival rate of these patients can reach more than 95%, and some of them can achieve a complete recovery [3, 4]. In contrast, the median survival of those at an advanced stage (stage-IV) is less than 12 months despite systematic treatment [5]. Hence, timely identification of early gastric cancer (EGC) is of essence to the prognosis of the patients.

Endoscopy is a prevalently used approach in clinical screening for gastrointestinal malignancies, and the identification of EGC depends greatly on endoscopic biopsy. Despite its high sensitivity and capability of identifying most of the cases, there is still a considerable omission diagnostic rate [6]. It is reported that the omission diagnostic rate of upper gastrointestinal malignancies reaches 15% in Western populations, which can be over 25% in Eastern countries such as Japan [7–9]. Endoscopy-based diagnosis relies largely on the image quality and endoscopists’ skill level of skill. An obscure image could easily misguide endoscopists to take the mucosal lesions of EGC for chronic atrophic gastritis [10] and the skill of endoscopists requires training and practicing for a long time [8]. In China, due to the large population base, severe imbalance of regional medical development, and uneven levels of doctors, the detection rate of EGC is not ideal. According to reports [11, 12], the detection rate of EGC in China is less than 5%, and the rate of missed diagnose under endoscopy is about 10%, which is obviously unfavorable to the prognosis of patients. In addition, the identification of EGC in gastroscopy mainly relies on the visual diagnosis and empirical judgment of doctors, which also poses a huge challenge to the accurate detection of EGC. Thus, there is an urgent need for effective approaches that can assist clinicians in endoscopic diagnosis and improve the diagnostic rate of EGC.

Machine learning (ML)-based endoscopy for EGC diagnosis has currently attracted extensive attention in clinical settings [13–15]. Deep learning (DL) methods based on convolutional neural network (CNN) exhibits great advantages in image recognition, segmentation, and feature extraction. Several studies have confirmed that it can be an auxiliary way to improve the accuracy of cancer diagnosis [16, 17]. However, it has diverse algorithms, and there is significant heterogeneity among different ML models. Even for the same ML model combined with different predictors, the diagnostic effect may vary. Therefore, ML can be a potential tool assisting in the diagnosis of EGC, while its performance lacks evidence-based support. Thereby, this systematic review and meta-analysis was performed to appraise the performance of ML-based endoscopy for EGC diagnosis, to provide evidence to update artificial intelligence (AI) tools in this field.

## Methods

We conduct this study in strict accordance with the PRISMA 2020 statement [18]. The protocol of this study has been registered on PROSPERO (registration No. CRD42022374248).

### Selection criteria

#### Inclusion criteria

- Types of participants: Adult EGC patients whose baseline characteristics and image information were recorded
- Types of study: Randomized controlled trial (RCT), case–control study, cohort study, nested case–control study, and case-cohort study
- Constructed a completed ML-based model for EGC diagnosis
- With or without the process of external validation. In ML research, it is difficult to conduct independent external validation due to limited conditions, so validation methods such as K-fold cross-validation or leave-one-out method are utilized. However, we cannot ignore the contributions that these studies have made, as we need to consider overfitting from the perspective of evidence-based medicine. Therefore, these articles were also included
- Studies using different ML models based on a same data set. In certain publicly authoritative datasets, different ML models have been developed, which were also included
- Reported and published in English

#### Exclusion criteria

- Other types of study, such as meta-analysis, review, guideline, and expert comments
- Only performed analysis for the risk factors, with no ML-based model completely constructed
- Lacked the following outcome measures: sensitivity (SEN), specificity (SPE), receiver operator characteristic curve (ROC), calibration curve, c-index, accuracy, precision rate, recovery rate, confusion matrix, diagnostic fourfold table, and F1 score
- Assessed the accuracy using univariate analysis

### Search strategy

A comprehensive electronic search was implemented up to September 25, 2022, in PubMed, Embase, Cochrane Library, and Web of Science. The strategy was designed based on Medical Subject Headings (MeSH) and free words. No restrictions were set to region and language.

### Study screening and data extraction

We used Endnote X9 for the management of the retrieved papers. Following the duplicate-checking, potentially eligible articles were screened by browsing the titles and abstracts, and we downloaded the full texts of potentially eligible articles. Studies that met the pre-set eligibility criteria were included after reading the full texts. A pre-designed form was adopted for extracting the data, which contained the following: title, author, publication date, nationality, study type, EGC cases, total cases, images of EGC, total images, EGC cases in training set, total cases in training set, images of EGC in training set, total images in training set, EGC cases in validation set, total cases in validation set, images of EGC in validation set, total images in validation set, model type, variables for model construction, and comparisons with clinicians. The above processes were completed independently by two reviewers (SYH and MB), and their results were cross-checked. Ant disagreements among them were addressed by a third reviewer (FSJ).

### Quality assessment

Quality Assessment of Diagnostic Accuracy Studies-2 (QUADAS-2) [19] was applied for the evaluation of the risk of bias. QUADAS-2 contains the following 4 aspects: patient selection, index test, reference standard, and flow and timing. Each domain includes several items that could be filled as “yes,” “no,” or “uncertain,” corresponding “low,” “high,” and “unclear” risk of bias, respectively. If all items in a domain are filled as “yes,” this domain would be graded as “low” risk of bias. If one item in a domain is filled as “no,” there would be potential bias, and the risk should be assessed according to the established guideline. “Unclear” refers to no detailed information provided in the study, which makes it difficult for reviewers to assess its risk of bias. The above processes were completed independently by the same two reviewers, and their results were cross-checked. Any disagreements among them were addressed by a third reviewer (FSJ).

### Statistical analysis

We used a bivariant mixed-effect model for meta-analysis. The model takes into account both fixed- and random-effects models and better handles heterogeneity across studies and the correlation between SEN and SPE, making the results more robust and reliable [20, 21]. The number of true positive (TP), false positive (FP), true negative (TN), and false negative (FN) cases in original studies were needed, while we could only obtain the SEN and SPE from several studies instead of the above information. Given this situation, we used the SEN and SPE in combination with EGC cases and total cases to calculate TP, FP, FN, and TN. Some studies only provided the ROC. In this case, we adopted Origin based on the optimal Youden index to extract the SEN and SPE from the ROC and subsequently calculated TP, FP, TN, and FN. The outcome variables in the bivariant mixed-effect model contained the SEN and SPE as well as the negative likelihood ratio (NLR), positive likelihood ratio (PLR), diagnostic odds ratio (DOR), and 95% confidence intervals (95%CI). Summarized ROC was produced and the area under the curve was computed. Deek’s funnel plot was utilized for publication bias assessment.

Subgroup analysis was processed based on the data sets (training set and validation set) and modeling variables (fixed images and dynamic videos). Moreover, we summarized the results of non-specialist clinicians/specialist clinicians, non-specialist clinicians/specialist clinicians with the assistance of ML, and video validation.

All the data analyses were done on Stata 15.0, and *p* < 0.05 implied statistical significance.

## Results

### Study selection

**Fig. 1:**
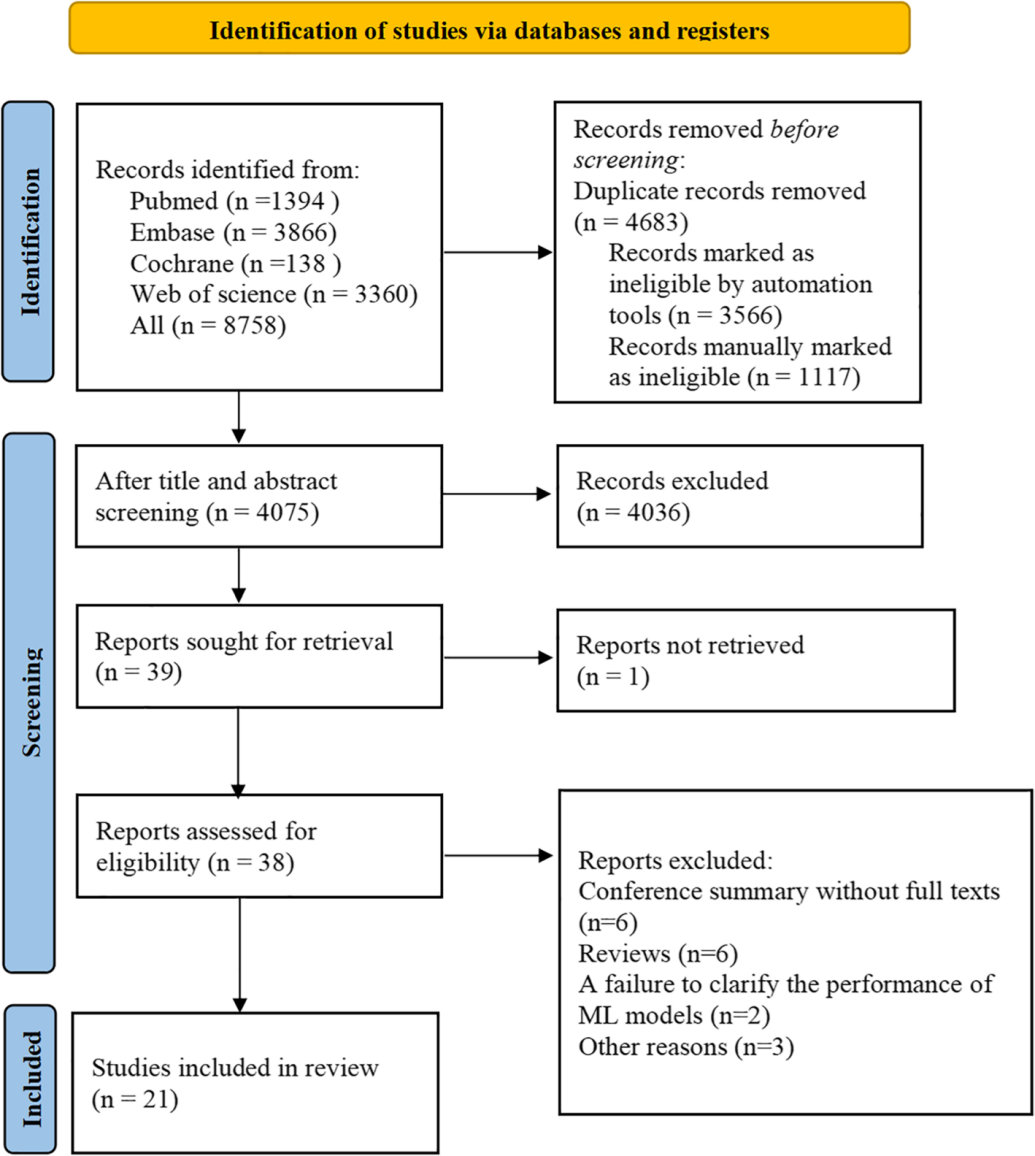
PRISMA 2020 flow diagram of the study selection process

There were 8758 articles retrieved through the literature search, of which 1394 were from PubMed, 3866 from Embase, 138 from Cochrane Library, and 3360 from Web of Science, and 4683 ineligible articles were removed due to duplication and other reasons. We screened the remaining 4075 articles through browsing their titles and abstracts, and 39 articles preliminarily met the inclusion criteria. Among these 39 articles, the full texts of 1 study could not be obtained, and full texts of the other 38 were read. After excluding conference summaries, reviews, studies with the full texts unavailable, and studies for which the diagnostic performance of the ML models could not be assessed, 21 articles were finally included. The flow diagram of study selection is presented in Fig. 1, and the detailed search strategies are shown in Table S1.

### Characteristics of the included articles

Twenty-one studies were included [13, 22–41], of which 14 studies [13, 22, 24, 26–35, 41] were conducted in China and 7 studies [23, 25, 36–40] were in Japan. There were 9 multi-centric studies [22, 24, 27–30, 34, 35, 41] and 4 prospective studies [13, 27, 30, 34]. There were 16,074 participants involved, and 454,528 endoscopic images were obtained, of which 97,950 images involved EGC. Among the included studies, 7 studies [13, 24, 30, 33–35, 39] performed real-time training or validation for ML-based models in videos, and 11 studies [24, 27, 29, 30, 32–38] provided comparisons for the diagnostic performance of the ML-based models with that of clinicians. We roughly divided those clinicians into specialists and non-specialists according to their working experience and the number of times of performing endoscopy yearly. The involving ML models were as follows: VGG-16, ResNet50, VGG-19, SVM, PLS-DA, ResNet34, DeepLabv3, GoogLeNet, EfficientDet, Darknet-53, ResNet101, and SSD. Detailed study characteristics are presented in Table S2.

### Quality assessment

**Fig. 2:**
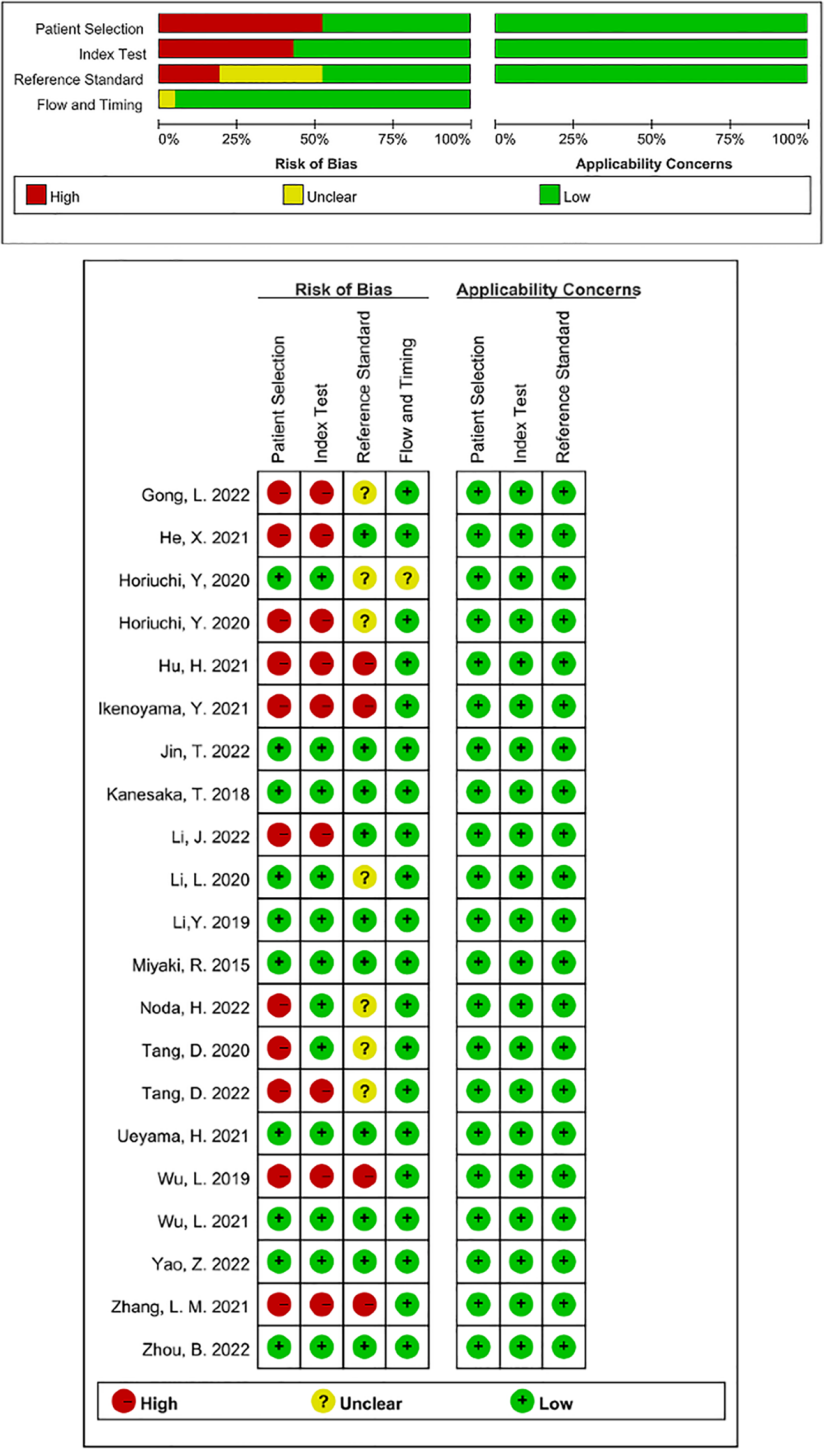
Risk of bias and clinical applicability assessment of included studies by QUADAS-2

By using QUADAS-2, the included studies were generally graded as high quality. Detailed results of the risk of bis assessment are exhibited in Fig. 2.

### Results of meta-analysis

#### Diagnostic performance of ML models in the image training set

**Fig. 3:**
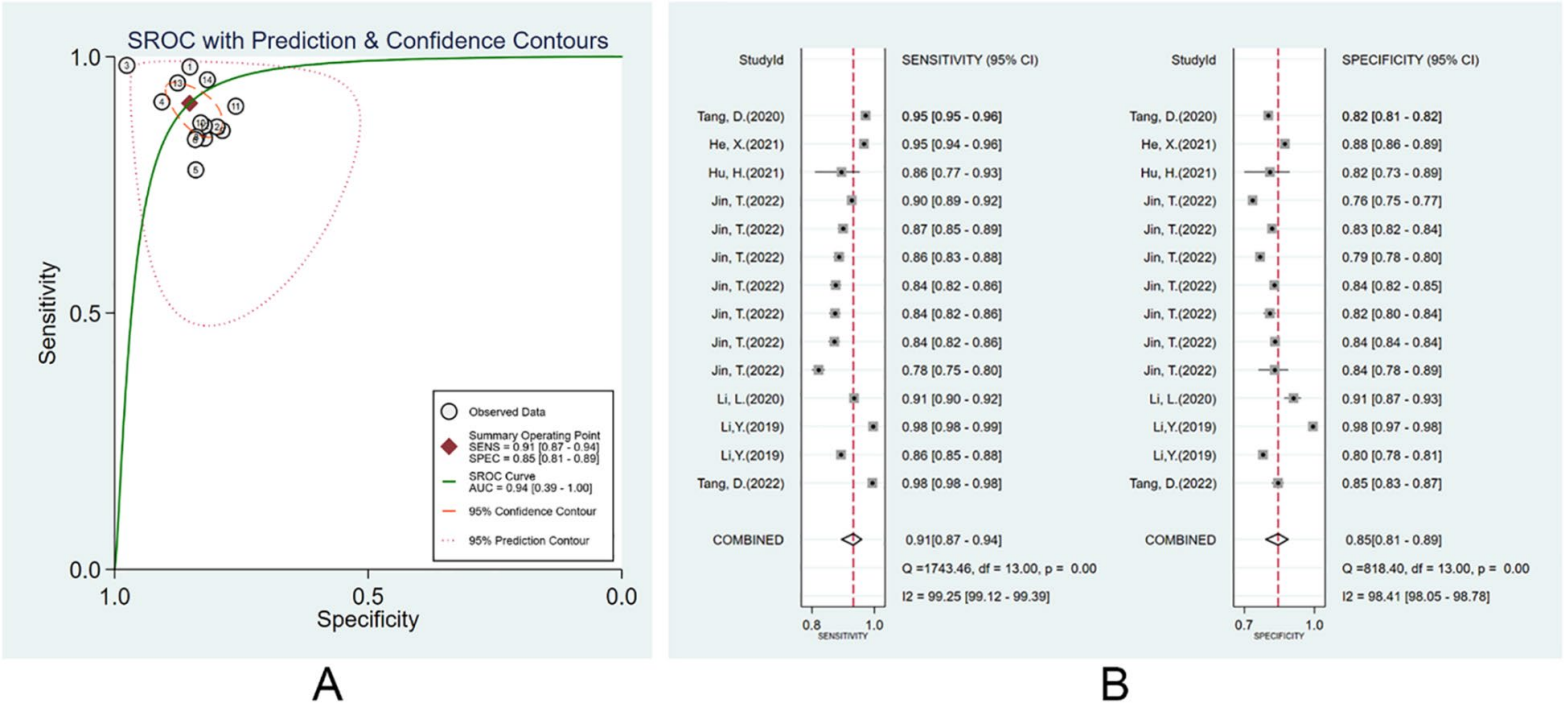
Diagnostic performance of the ML models in image training set. **A** SROC; **B** forest plot of pooled SEN and SPE

There were 7 studies [24, 26–30, 35] that trained endoscopic image-based ML models for EGC diagnosis. The pooled AUC, SEN, and SPE were 0.94 (95% CI: 0.39–1.00), 0.91 (95% CI, 0.87–0.94), and 0.85 (95% CI: 0.81–0.89) (Fig. 3A, B). The PLR, NLR, and DOR were 6.2 (95% CI: 4.6–8.2), 0.11 (95% CI: 0.07–0.16), and 58 (95% CI: 29–114), respectively. No evident publication bias was found (*p* = 0.51). More details are provided in Supplementary Fig. 1.

#### Diagnostic performance of ML models in the image validation set

**Fig. 4:**
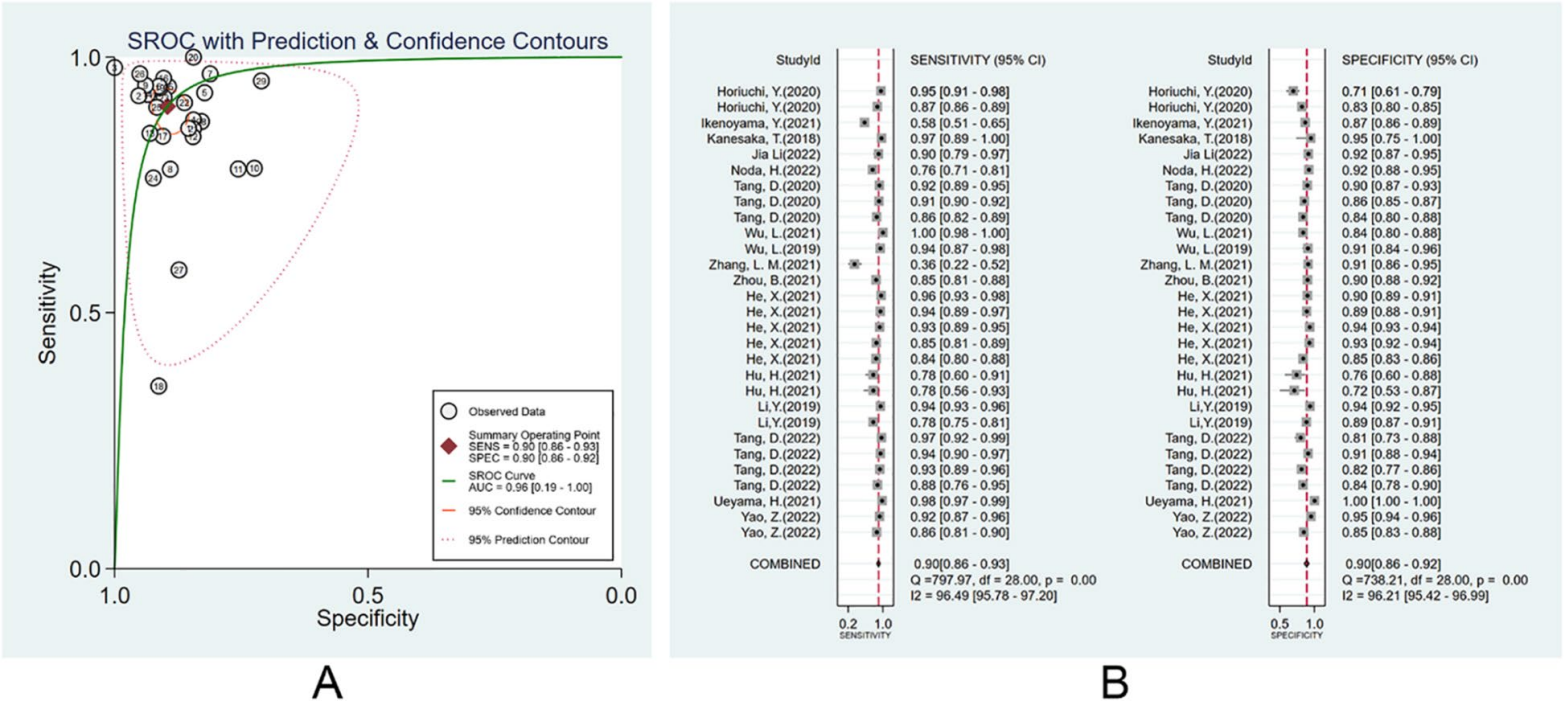
Diagnostic performance of the ML models in image validation set. **A** SROC; **B** forest plot of pooled SEN and SPE

There were 17 studies [13, 22–24, 26, 29–40] that validated the performance of the ML models for diagnosing EGC, and 6 of them [22, 24, 26, 29, 30, 35] had included more than 1 set of data. The pooled AUC, SEN, and SPE were 0.96 (95% CI: 0.19–1.00), 0.90 (95% CI: 0.86–0.93), and 0.90 (95% CI: 0.86–0.92) (Fig. 4A, B). The PLR, NLR, and DOR were 8.7 (95% CI: 6.6–11.4), 0.11 (95% CI: 0.08–0.15), and 80 (95% CI: 47–138), respectively. No evident publication bias was noted (*p* = 0.84). More details are provided in Supplementary Fig. 2.

#### Diagnostic performance of clinicians

**Fig. 5:**
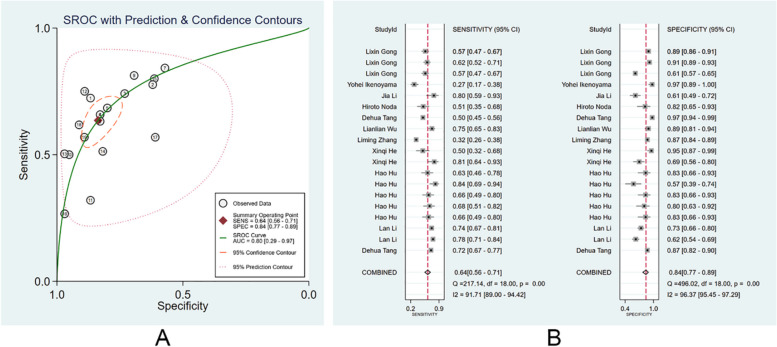
Diagnostic performance of non-specialist clinicians in the diagnosis of EGC through endoscopic images. **A** SROC; **B** forest plot of pooled SEN and SPE

**Fig. 6:**
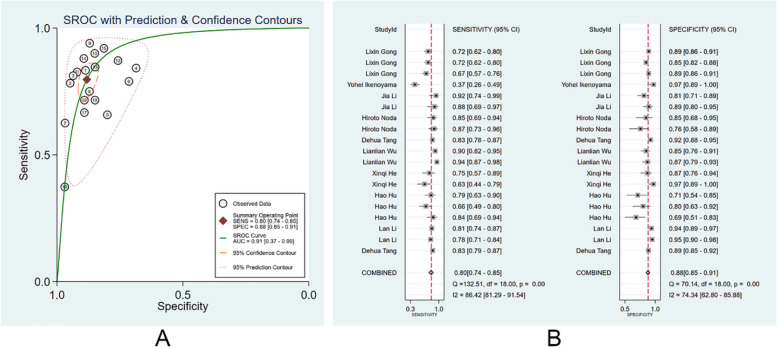
Diagnostic performance of specialist clinicians in the diagnosis of EGC by endoscopic images. **A** SROC; **B** forest plot of pooled SEN and SPE

We divided those clinicians into specialists and non-specialists according to their working experience and the number of times of endoscopy performed. There were 72 non-specialist clinicians, and the pooled AUC, SEN, and SPE were 0.80 (95% CI: 0.29–0.97), 0.64 (95% CI: 0.56–0.71), and 0.84 (95% CI: 0.77–0.89) (Fig. 5A, B). The PLR, NLR, and DOR were 4 (95% CI: 2.9–5.3), 0.44 (95% CI: 0.37–0.52), and 9 (95% CI: 6–13), respectively. No evident publication bias was noticed (*p* = 0.94). There were 76 specialist clinicians, and the pooled AUC, SEN, and SPE were 0.91(95% CI: 0.37–0.99), 0.80 (95% CI: 0.74–0.85), and 0.88 (95% CI: 0.85–0.91) (Fig. 6A, B). The PLR, NLR, and DOR were 6.7 (95% CI: 5.4–8.4), 0.23 (95% CI: 0.18–0.30), and 29 (95% CI: 21–41), respectively. No evident publication bias existed (*p* = 0.27). More details are provided in Supplementary Figs. 3 and 4.

#### Diagnostic performance of clinicians with the assistance of ML models

**Fig. 7:**
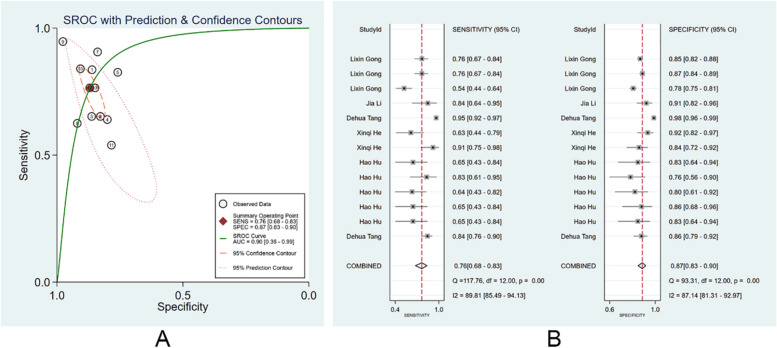
Diagnostic performance of non-specialist clinicians with assistance of the machine learning models in the diagnosis of EGC by endoscopic images. **A** SROC; **B** forest plot of pooled SEN and SPE

**Fig. 8:**
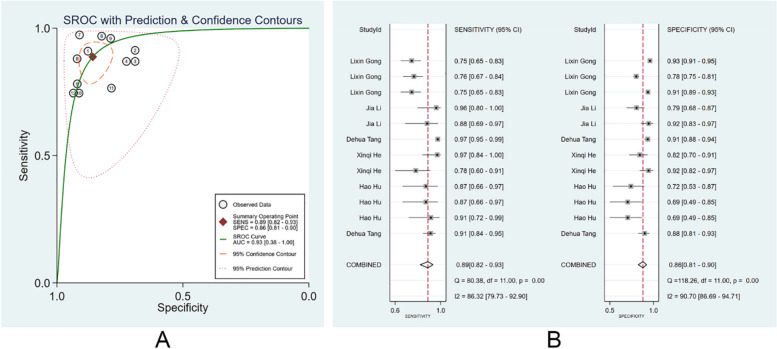
Diagnostic performance of specialist clinicians with assistance of the machine learning models in the diagnosis of EGC by endoscopic images. **A** SROC; **B** forest plot of pooled SEN and SPE

There were 6 studies [13, 24, 29, 30, 35, 41] reporting the performance of clinicians in diagnosing EGC with the assistance of ML models. We also divided these clinicians into specialist clinicians and non-specialist clinicians. There were 16 specialist clinicians and 12 non-specialist clinicians. With the assistance of the ML models, the pooled AUC, SEN, and SPE of non-specialist clinicians were 0.90 (95% CI: 0.36–0.99), 0.76 (95% CI: 0.68–0.83), and 0.87 (95% CI: 0.83–0.90), (Fig. 7A, B). The PLR, NLR, and DOR were 6 (95% CI: 4.1–8.3), 0.27 (95% CI: 0.19–0.38), and 21 (95% CI:11–43). No evident publication bias was existed (*p* = 0.10). With the assistance of the ML models, the pooled AUC, SEN, and SPE of specialist clinicians were 0.93 (95% CI: 0.38–1.00), 0.89 (95% CI: 0.82–0.93), and 0.86 (95% CI: 0.81–0.90), respectively (Fig. 8A, B). The PLR, NLR, and DOR were 6 (95% CI: 4.6–8.6), 0.13 (95% CI: 0.08–0.21), and 48 (95% CI: 26–87), respectively. No evident publication bias was noticed (*p* = 0.22). More details are provided in Supplementary Figs. 5 and 6.

#### Diagnostic performance of ML models in the video validation set

**Fig. 9:**
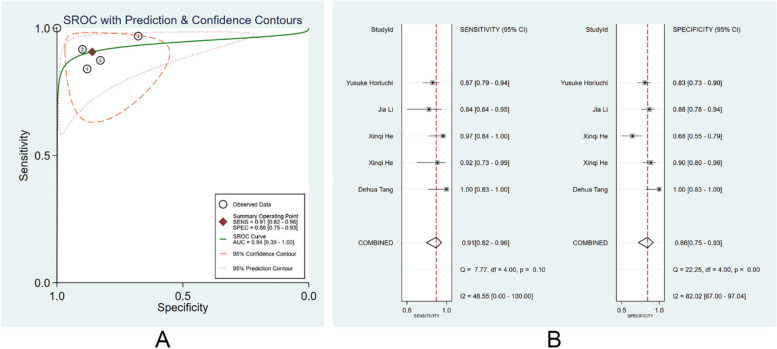
Performance of ML models in the diagnosis of EGC in video validation set. **A** SROC; **B** forest plot of pooled SEN and SPE

There were 4 studies [13, 24, 30, 39] that validated the diagnostic performance of ML models in real-time videos. The pooled AUC, SEN, and SPE were 0.94 (95% CI: 0.39–1.00), 0.91 (95% CI: 0.82–0.96), and 0.86 (95% CI: 0.75–0.93) (Fig. 9A, B). The PLR, NLR, and DOR were 6 (95% CI: 3.5–12.1), 0.11 (95%CI: 0.05–0.22), and 60 (95% CI: 20–176), respectively. No evident publication bias existed (*p* = 0.08). More details are provided in Supplementary Fig. 7.

#### Diagnostic performance of clinicians in the video validation set

**Fig. 10:**
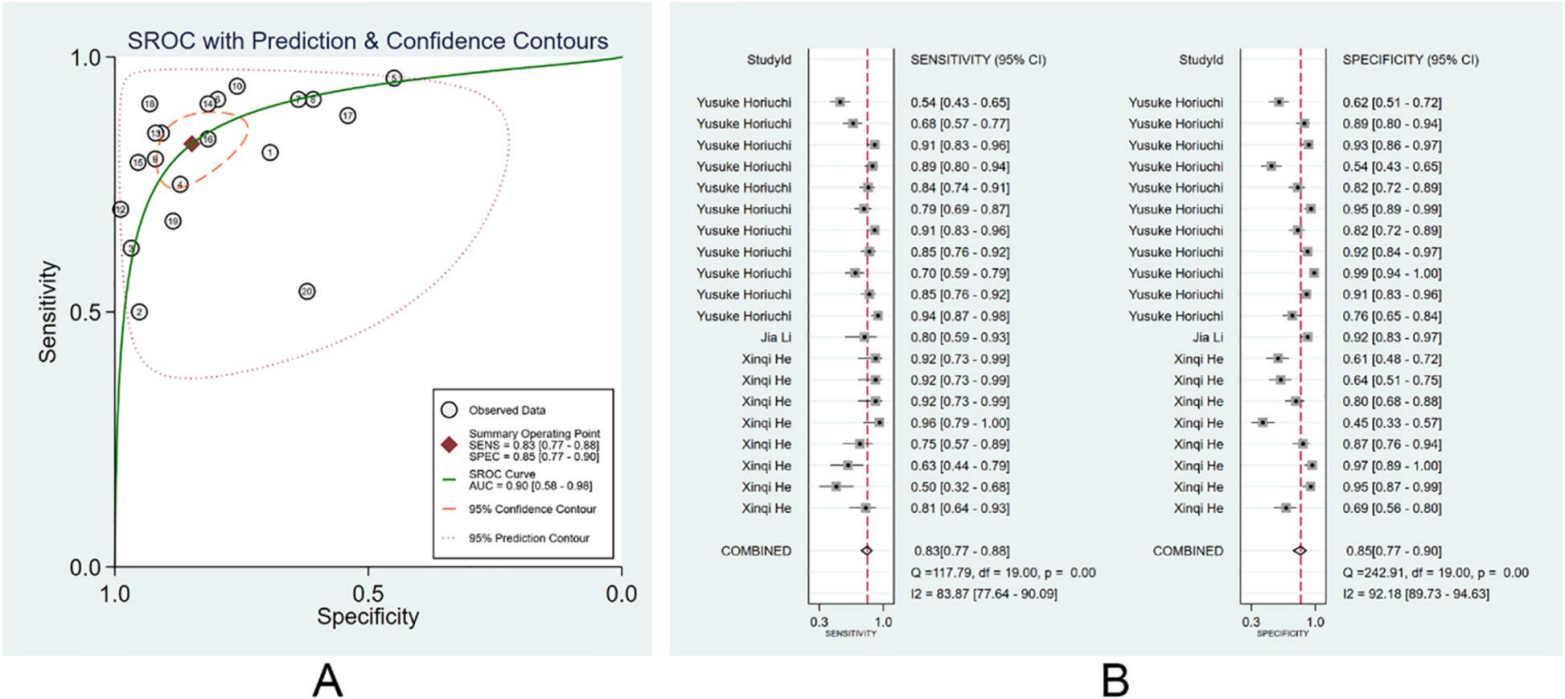
Performance of clinicians in the diagnosis of EGC in video validation set. **A** SROC; **B** forest plot of pooled SEN and SPE

There were 3 studies [13, 30, 39] that validated the performance of clinicians (*n* = 20) in the diagnosis of EGC in real-time videos. The pooled AUC, SEN, and SPE were 0.90 (95% CI: 0.58–0.98), 0.83 (95% CI: 0.77–0.88), and 0.85 (95% CI: 0.77–0.90) (Fig. 10A, B). The PLR, NLR, and DOR were 5 (95% CI: 3.6–8.2), 0.20 (95% CI: 0.15–0.27), and 27 (95% CI: 17–44), respectively. No evident publication bias was noticed (*p* = 0.51). More details are provided in Supplementary Fig. 8.

## Discussion

In this study, we systematically searched articles regarding the application of ML for the diagnosis of EGC, assessed the application value of image-based ML models for EGC diagnosis, and compared the performance of these models with clinicians of different skill levels. Moreover, we assessed the diagnostic performance of ML models in real-time videos. The analysis results revealed that ML models would be of greater performance in diagnosing endoscopic images than clinicians (including specialists and non-specialists), and the diagnostic performance of non-specialist clinicians could be improved to the level of the specialists with the assistance of ML models. ML models presented a remarkable performance in real-time video diagnosis, and the sensitivity and specificity were all higher than those of clinicians.

ML is a crucial part of artificial intelligence. It is composed of multiple disciplines and can learn and practice with a large amount of historical data to construct algorithm models that provide accurate prediction and assessment for the new data [42, 43], which refers to a process from experience summarizing to flexible use. ML technique has been extensively employed in screening gastrointestinal malignancies, mainly in assisting endoscopic diagnosis, automatic pathological examination, and tumor invasion depth detection, and has produced desired results. [44] Chang et al. [45] reviewed the diagnostic performance of endoscopic image-based ML for early esophageal cancer. The AUC, SEN, and SPE were 0.97 (95% CI 0.95–0.99), 0.94 (95% CI, 0.89–0.96), and 0.88 (95% CI, 0.76–0.94). Jiang et al. [46] included 16 articles and found that the AUC, SEN, and SPE of AI-assisted EGC diagnosis were 0.96 (95% CI: 0.94–0.97), 86% (95% CI: 77–92%), and 93% (95% CI: 89–96%). However, Luo et al. [47] included 15 articles and reported the pooled AUC, SEN, and SPE of endoscopic images-based AI in the detection of EGC were 0.94, 0.87 (95% CI: 0.87–0.88), and 0.88 (95% CI: 0.87–0.88). Variances in the diagnostic performance of ML models among different studies indicate significant heterogeneity among different models. ML models can have overfitting or underfitting problems when dealing with specific datasets, which can limit their application and generalization [48, 49]. Thereby, we strictly differentiated between the results of the training set and validation set, which could help us to analyze whether ML models are at risk of overfitting and underfitting and to reflect whether there are any challenges in the goodness-of-fit of the existing ML models from an evidence-based medicine perspective. Fortunately, our results were not overfitting or underfitting. Additionally, validating the model performance in different datasets with adequate external validation is necessary to improve the model and increase its reliability and application [50]. There is a current lack of articles comparing the performance of ML-based models with clinicians of different skill levels and clinicians with the assistance of ML models in EGC diagnosis as well as studies validating the diagnostic performance of ML models in real-time videos. Our study has filled the gap.

According to our study, the mainstream ML method is CNN. CNN is among the most typical DL models, which includes multiple algorithm models such as VGG, GoogleNet, ResNet, and DenseNet [51]. It is of excellent image recognition and classification ability and has been widely applied in endoscopic image-based diagnosis [27, 52]. Fang et al. [53] revealed the AUC, SEN, and SPE of CNN in the endoscopic image-based GC diagnosis were 0.89, 0.83, and 0.94. Md Mohaimenul Islam et al. [54] revealed that the SROC and SEN of the CNN model in EGC diagnosis were 0.95 and 0.89, respectively. Among the articles included, only 2 articles [25, 26] used conventional ML methods (SVM). Miyaki, R et al. [25] discovered the mean SVM output-value of the cancer lesion was 0.846 ± 0.220, which was evidently higher than that of the reddened lesions (0.381 ± 0.349) and surrounding tissues (0.219 ± 0.277). Yuanpeng Li et al. [26] elicited the SEN, SPE, and accuracy of SVM in diagnosing EGC were all over 90%, indicating its good application value. However, conventional ML methods such as SVM have more limitations compared to DL models. The former relies on experienced experts to manually design the image features, requires multiple calculations to obtain the best truncation value, and yields poor performance in processing large-scale data sets [44, 55, 56]. All of these problems impede the further development of conventional ML methods.

We observed, in this study, that ML-based models had a higher diagnostic sensitivity than clinicians. These models showed diagnostic performance as good as clinical specialists in both the images and videos. With the assistance of ML, the diagnostic sensitivity of non-specialists and specialists for EGC was significantly improved, while such an improvement was not observed in the specificity, and the specificity of ML-assisted specialists was slightly lower than the ML models. This indicated that the assistance of ML increased the specialists` misdiagnosis rate. Misdiagnosis caused by ML models in the process of image recognition is often attributed to the poor endoscopic image resolution leading to an abnormal mucosal background color, which could be induced by residual foam, blood, and food residues in the lesion site, and confusing tissue structures such as atrophic gastritis, intestinal metaplasia, and ulcers [29, 30]. ML models could interfere with clinical experts` judgment by presenting them with misidentified information, as reported by Tang et al. [24] In addition, in video diagnosis, the SROC, SEN, and SPE of ML models for EGC were 0.94 (95% CI: 0.39–1.00), 0.91 (95% CI: 0.82–0.96), and 0.86 (95% CI: 0.75–0.93), greater than that of clinicians: the SROC, SEN, and SPE were 0.90 (95% CI: 0.58–0.98), 0.83 (95% CI: 0.77–0.88), and 0.85 (95% CI: 0.77–0.90). By comparing the performance between ML models in EGC diagnosis in images and real-time videos, we found that video slightly outperformed image on SEN, with image vs. video at 0.90 vs. 0.91. And image slightly outperformed video on SROC (0.96 vs. 0.94) and SPE (0.9 vs. 0.86). However, this is not enough to clarify whose performance of ML models is better in images and real-time videos. Because only 4 papers validated the detection performance of ML models in real-time videos, with a significantly smaller sample size than images. Thus, more original studies are still needed to validate the diagnostic performance of ML models in real-time videos to better compare their performance. Indeed, video diagnostics also presents unique challenges [57, 58]. First, compared to images, videos contain dynamic and time-dependent information, which makes processing and analysis more difficult. Second, the training and inference of ML models usually require high-performance computers and many computational resources. Videos contain much frame and pixel information and thus require higher computation and equipment requirements. Finally, due to the specificity of the medical field, the use of ML models for cancer diagnosis may involve many complex regulatory and ethical issues. However, it is undeniable that ML-based models can serve as an adjuvant diagnostic approach for EGC, bringing effective help to clinicians in clinical practice, especially for non-specialists. It could improve their diagnostic performance to the level of specialists while reducing costs. The study demonstrates the feasibility of ML methods for EGC diagnosis, which facilitates the development of AI tools to provide diagnostic assistance to inexperienced clinicians and in areas where medical resources are scarce.

This study also has limitations. Firstly, most included articles were retrospective-design, and only few articles performed prospective validation for the constructed ML models. Retrospective studies may suffer from incomplete data collection, poor quality, and bias, which affect the generalizability of the findings [49, 50]. Therefore, the performance of ML models in EGC diagnosis needs to be validated by more prospective studies. Secondly, most included articles had excluded manually images of poor quality during the image selection process, which might cause an overestimated diagnostic performance of these models. The included images were also less likely to include all types of GC lesions that could be used as controls to EGC, making it difficult to conduct comprehensive training of the models, and their application was subsequently limited. In addition, ML models in most of the included studies were constructed with DL, and subgroup analysis for different types of ML (e.g., VGG-16, ResNet50, VGG-19) could not be performed owing to the limited included articles. Due to the limited number of ML methods, we also failed to conduct a more detailed subgroup analysis of different ML models (e.g., CNN, SVM). Lastly, the model construction in the included articles was mostly based on static endoscopic images, which is different from the real-time clinical operation scenarios. More original articles are needed to further validate the diagnostic performance of ML models in real-time videos.

## Conclusion

This meta-analysis demonstrates that ML-based diagnostic models have great performance in EGC diagnosis, with the sensitivity and specificity all higher than those of clinical specialists. It has great application prospects and can be used as an adjuvant approach to help clinicians make more accurate diagnoses.

### Supplementary Information

**Additional file 1: Supplementary Fig. 1.** Meta-analysis of the predictive accuracy of image-based machine learning models in diagnosis of early GC in the training cohort (**A**) Funnel plot for publication bias; (**B**) Heterogeneity box plot; (**C**) Clinical application nomogram. **Supplementary Fig. 2.** Meta-analysis of the predictive accuracy of image-based machine learning models in diagnosis of early GC in the validation cohort (**A**) Funnel plot for publication bias; (**B**) Heterogeneity box plot; (**C**) Clinical application nomogram. **Supplementary Fig. 3.** Meta-analysis of the predictive accuracy of non-specialist clinicians with assistance of endoscopic images in diagnosis of early GC (**A**) Funnel plot for publication bias; (**B**) Heterogeneity box plot; (**C**) Clinical application nomogram. **Supplementary Fig. 4.** Meta-analysis of the predictive accuracy of specialist clinicians with assistance of endoscopic images in the diagnosis of early GC (**A**) Funnel plot for publication bias; (**B**) Heterogeneity box plot; (**C**) Clinical application nomogram. **Supplementary Fig. 5.** Meta-analysis of non-specialist clinicians with assistance of the machine learning models in the diagnosis of early GC by endoscopic images (**A**) Funnel plot for publication bias; (**B**) Heterogeneity box plot; (**C**) Clinical application nomogram. **Supplementary Fig. 6.** Meta-analysis of the predictive accuracy of specialist clinicians with assistance of the machine learning models in the diagnosis of early GC by endoscopic images (**A**) Funnel plot for publication bias; (**B**) Heterogeneity box plot; (**C**) Clinical application nomogram. **Supplementary Fig. 7.** Meta-analysis of the predictive accuracy of machine learning models in diagnosis of early GC in the video validation cohort (**A**) Funnel plot for publication bias; (**B**) Heterogeneity box plot; (**C**) Clinical application nomogram. **Supplementary Fig. 8.** Meta-analysis of the predictive accuracy of clinicians in diagnosis of early GC in the video validation cohort (**A**) Funnel plot for publication bias; (**B**) Heterogeneity box plot; (**C**) Clinical application nomogram.

**Additional file 2: Table S1.** Literature search strategy. **Table S2.** Basic characteristics of the included literature.

## Data Availability

The data that support the findings of this study are available from the corresponding author upon reasonable request.

## Abbreviations

EGC: Early gastric cancer
ML: Machine learning
HP: Helicobacter pylori
AI: Artificial intelligence
DL: Deep learning
CNN: Convolutional neural network
RCT: Randomized controlled trial
MeSH: Medical Subject Headings
TP: True positive
FP: False positive
TN: True negative
FN: False negative
NLR: Negative likelihood ratio
PLR: Positive likelihood ratio
DOR: Diagnostic odds ratio
95%CI: 95% Confidence intervals
SROC: Summarized receiver operator characteristic
