# Supplementary material for: The value of machine learning approaches in the diagnosis of early gastric cancer: a systematic review and meta-analysis
Source: World J Surg Oncol. 2024 Feb 1;22:40. doi: 10.1186/s12957-024-03321-9 (PMC10832162; doi:10.1186/s12957-024-03321-9)
Supplement: Supplementary file 1 — Additional file 1: Supplementary Fig. 1. Meta-analysis of the predictive accuracy of image-based machine learning models in diagnosis of early GC in the training cohort (A) Funnel plot for publication bias; (B) Heterogeneity box plot; (C) Clinical application nomogram. Supplementary Fig. 2. Meta-analysis of the predictive accuracy of image-based machine learning models in diagnosis of early GC in the validation cohort (A) Funnel plot for publication bias; (B) Heterogeneity box plot; (C) Clinical application nomogram. Supplementary Fig. 3. Meta-analysis of the predictive accuracy of non-specialist clinicians with assistance of endoscopic images in diagnosis of early GC (A) Funnel plot for publication bias; (B) Heterogeneity box plot; (C) Clinical application nomogram. Supplementary Fig. 4. Meta-analysis of the predictive accuracy of specialist clinicians with assistance of endoscopic images in the diagnosis of early GC (A) Funnel plot for publication bias; (B) Heterogeneity box plot; (C) Clinical application nomogram. Supplementary Fig. 5. Meta-analysis of non-specialist clinicians with assistance of the machine learning models in the diagnosis of early GC by endoscopic images (A) Funnel plot for publication bias; (B) Heterogeneity box plot; (C) Clinical application nomogram. Supplementary Fig. 6. Meta-analysis of the predictive accuracy of specialist clinicians with assistance of the machine learning models in the diagnosis of early GC by endoscopic images (A) Funnel plot for publication bias; (B) Heterogeneity box plot; (C) Clinical application nomogram. Supplementary Fig. 7. Meta-analysis of the predictive accuracy of machine learning models in diagnosis of early GC in the video validation cohort (A) Funnel plot for publication bias; (B) Heterogeneity box plot; (C) Clinical application nomogram. Supplementary Fig. 8. Meta-analysis of the predictive accuracy of clinicians in diagnosis of early GC in the video validation cohort (A) Funnel plot for publication bias; [file 12957_2024_3321_MOESM1_ESM.docx]

**A**

**
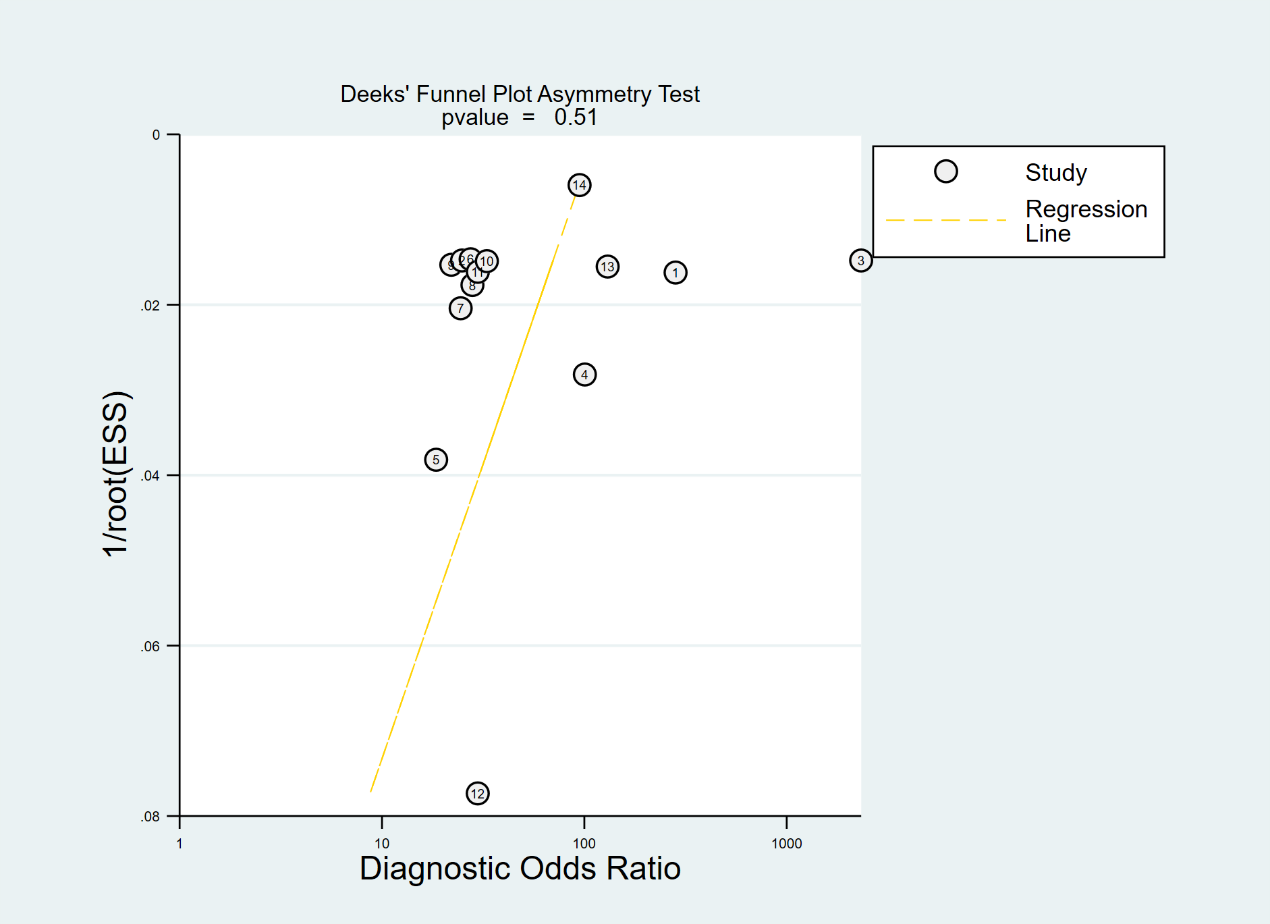
**

**B**

**
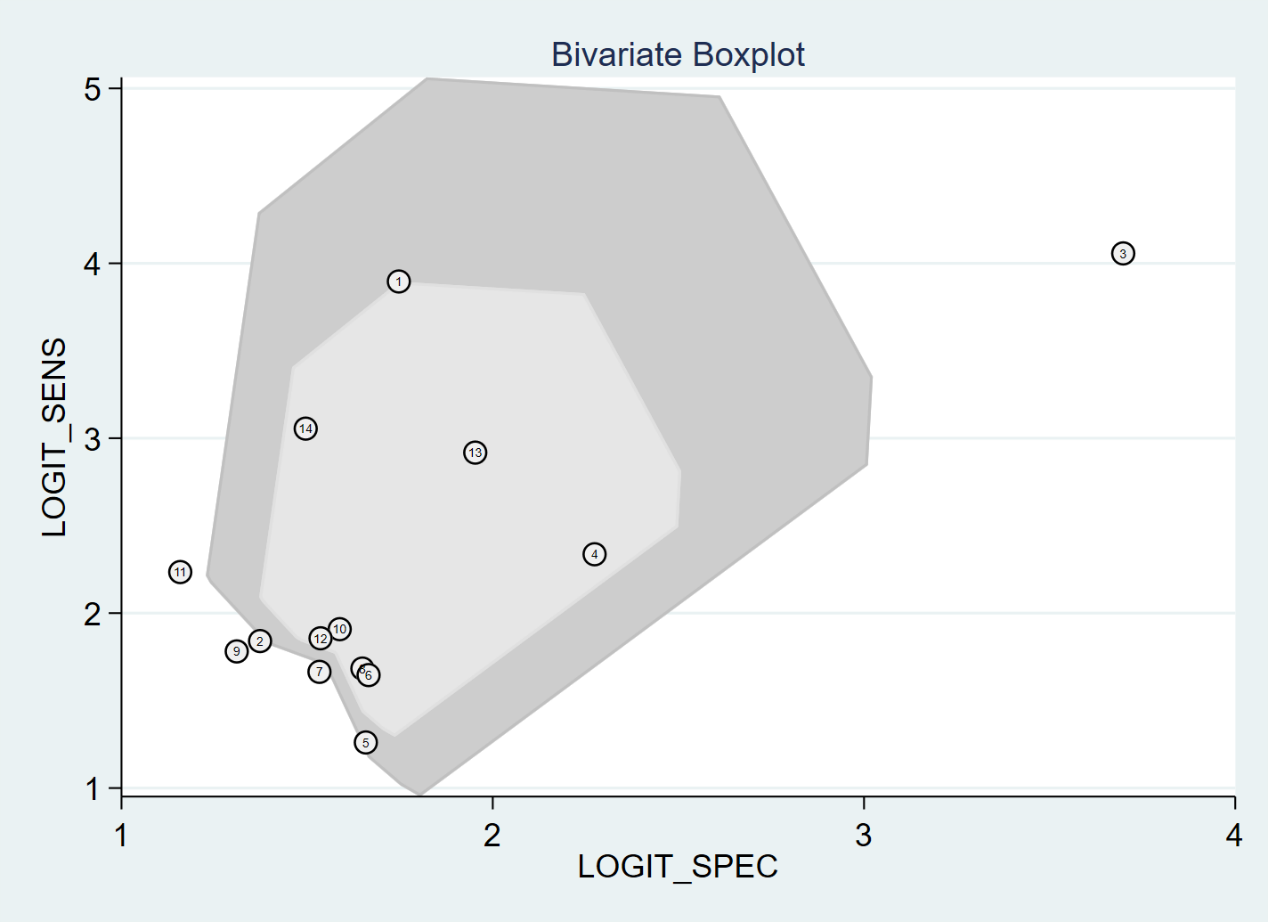
**

**C**


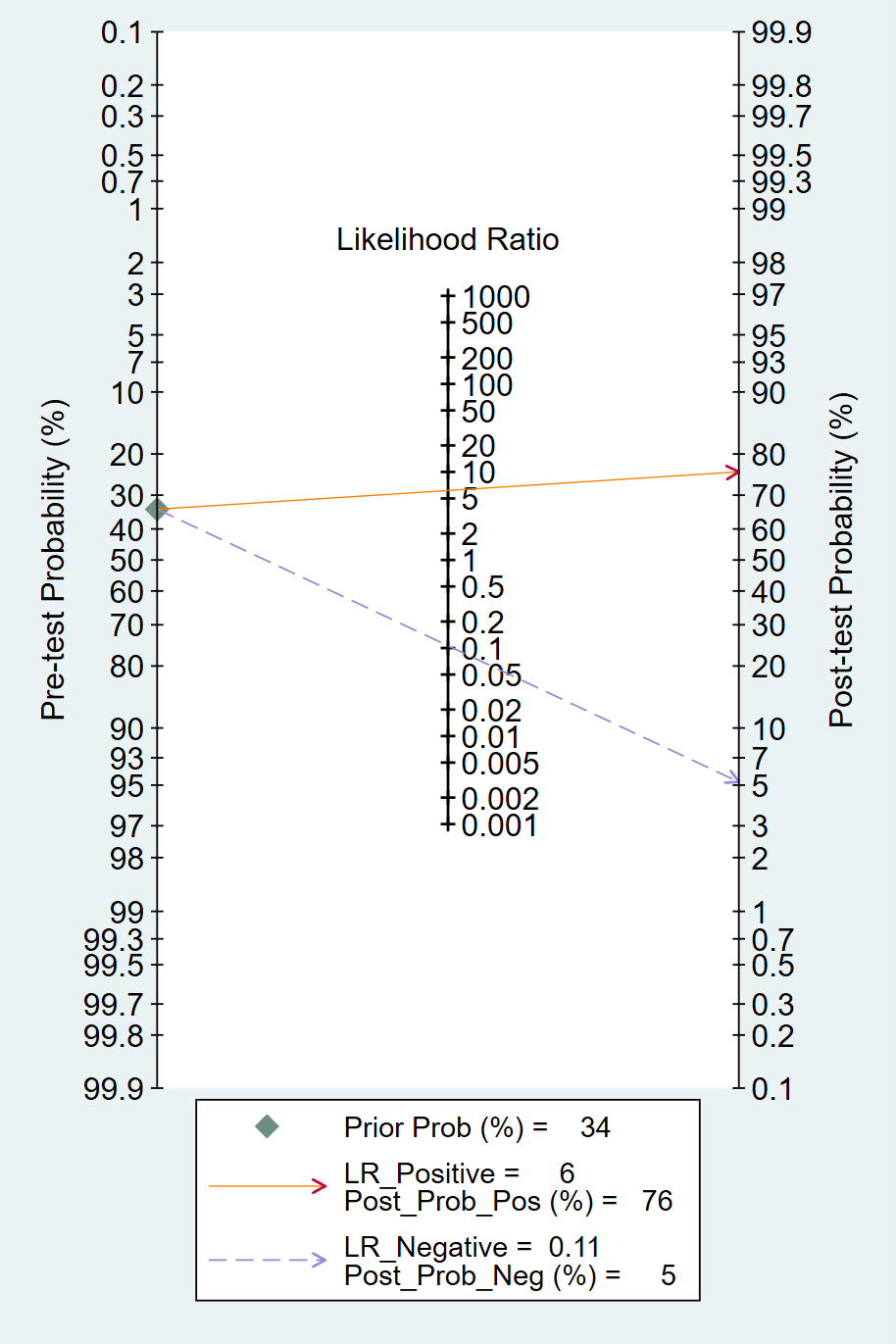


**Supplementary Figure 1** Meta-analysis of the predictive accuracy of image-based machine learning models in diagnosis of early GC in the training cohort (**A**) Funnel plot for publication bias; (**B**) Heterogeneity box plot; (**C**) Clinical application nomogram

**A**

**
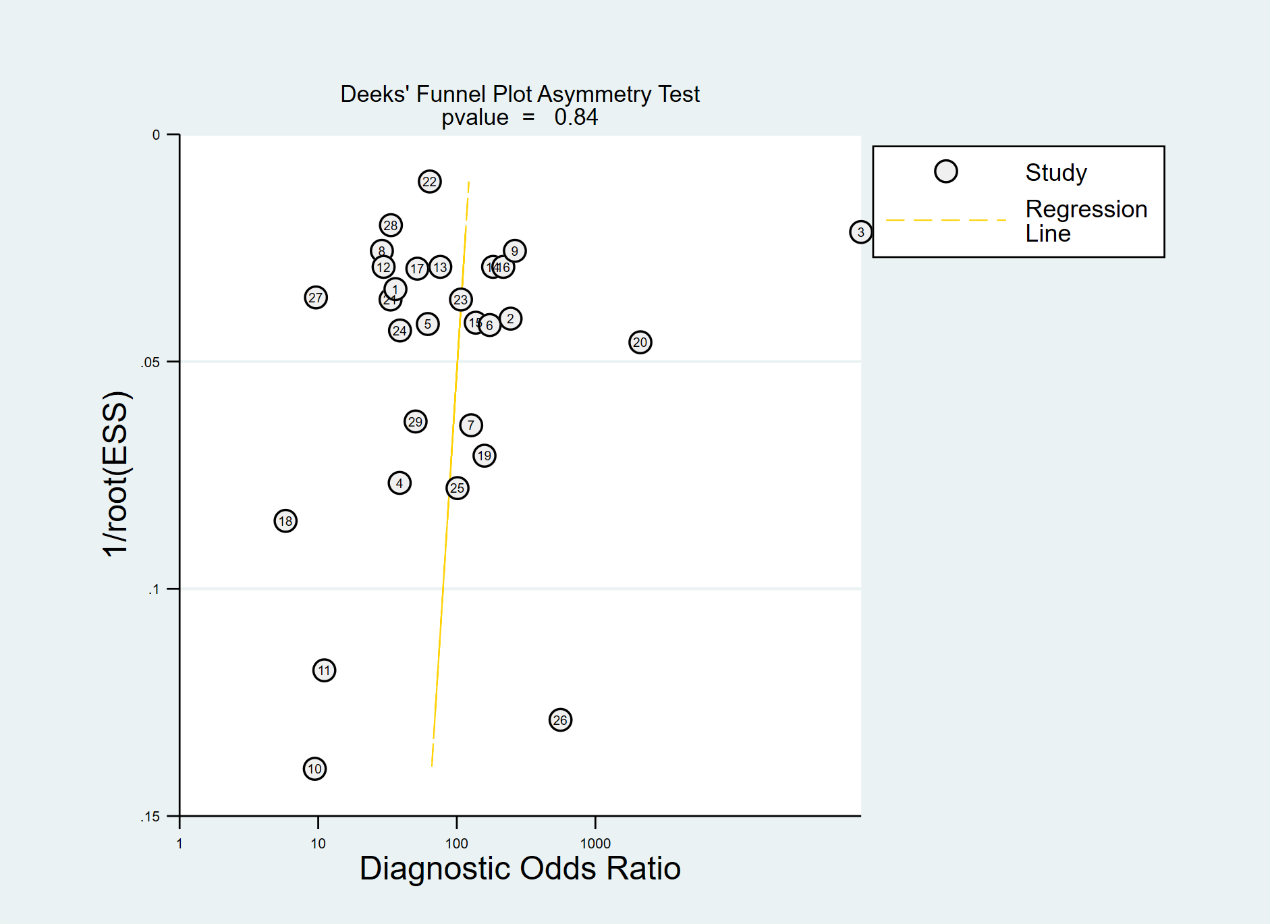
**

**B**

**
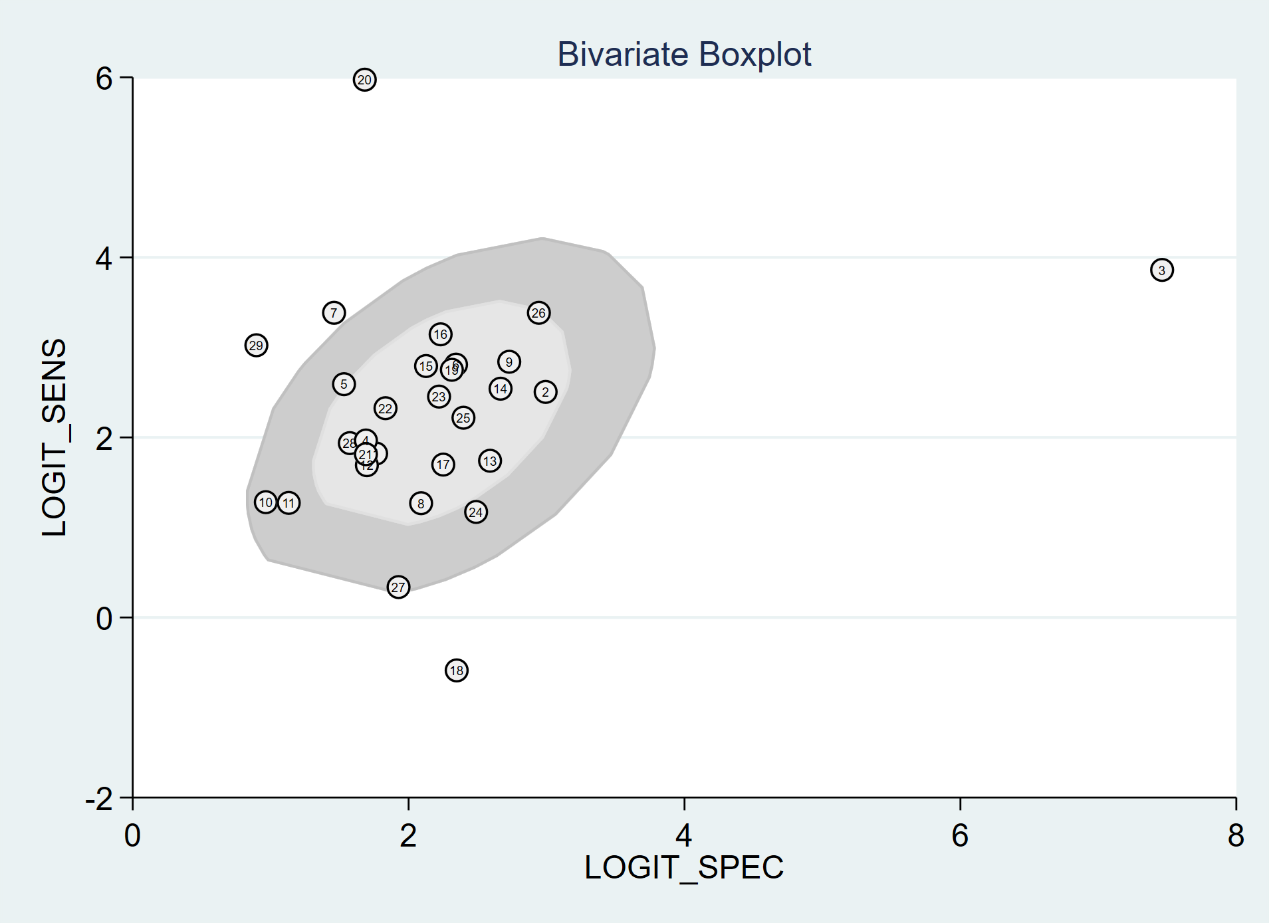
**

**C**


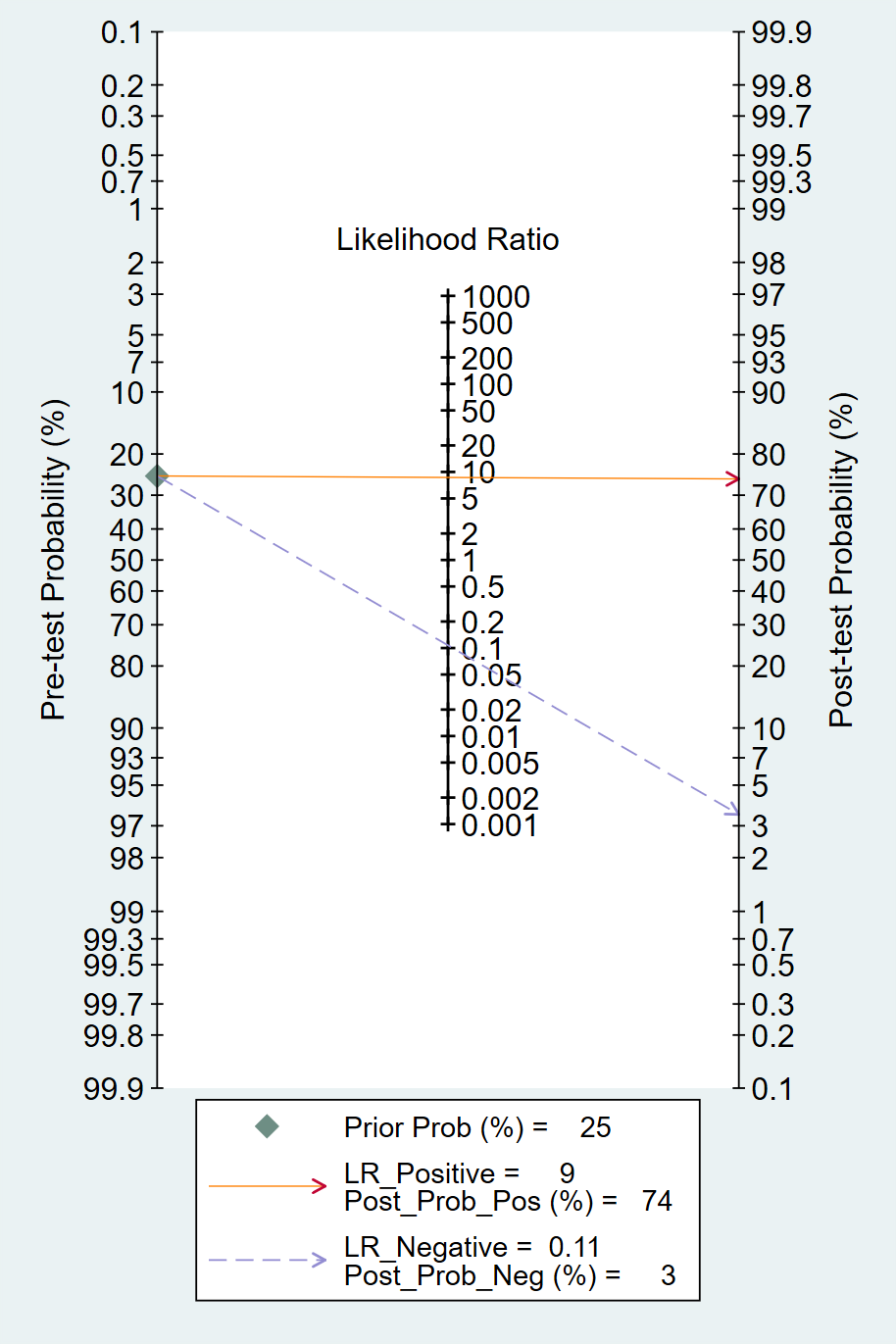


**Supplementary Figure 2.** Meta-analysis of the predictive accuracy of image-based machine learning models in diagnosis of early GC in the validation cohort (**A**) Funnel plot for publication bias; (**B**) Heterogeneity box plot; (**C**) Clinical application nomogram

**A**

**
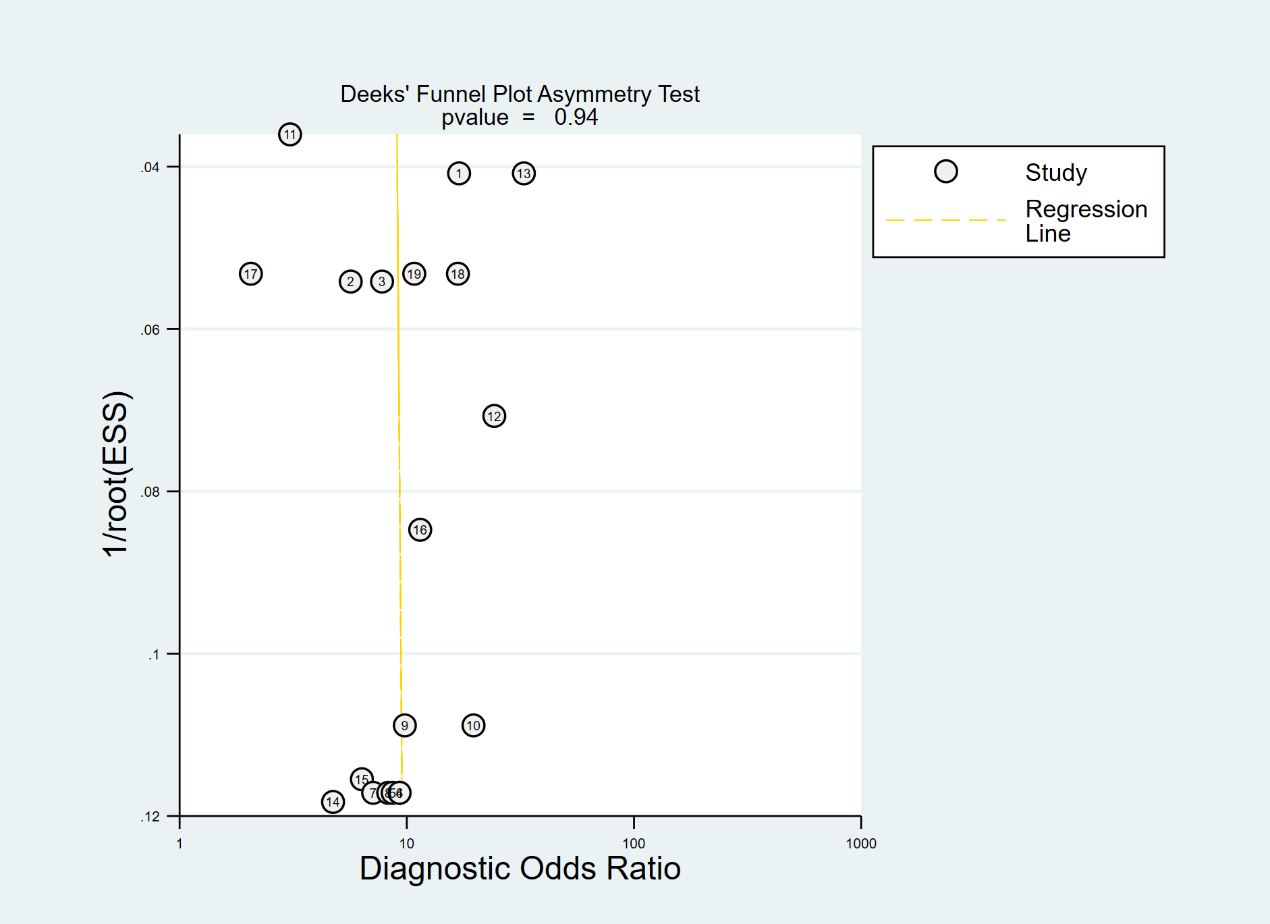
**

**B**

**
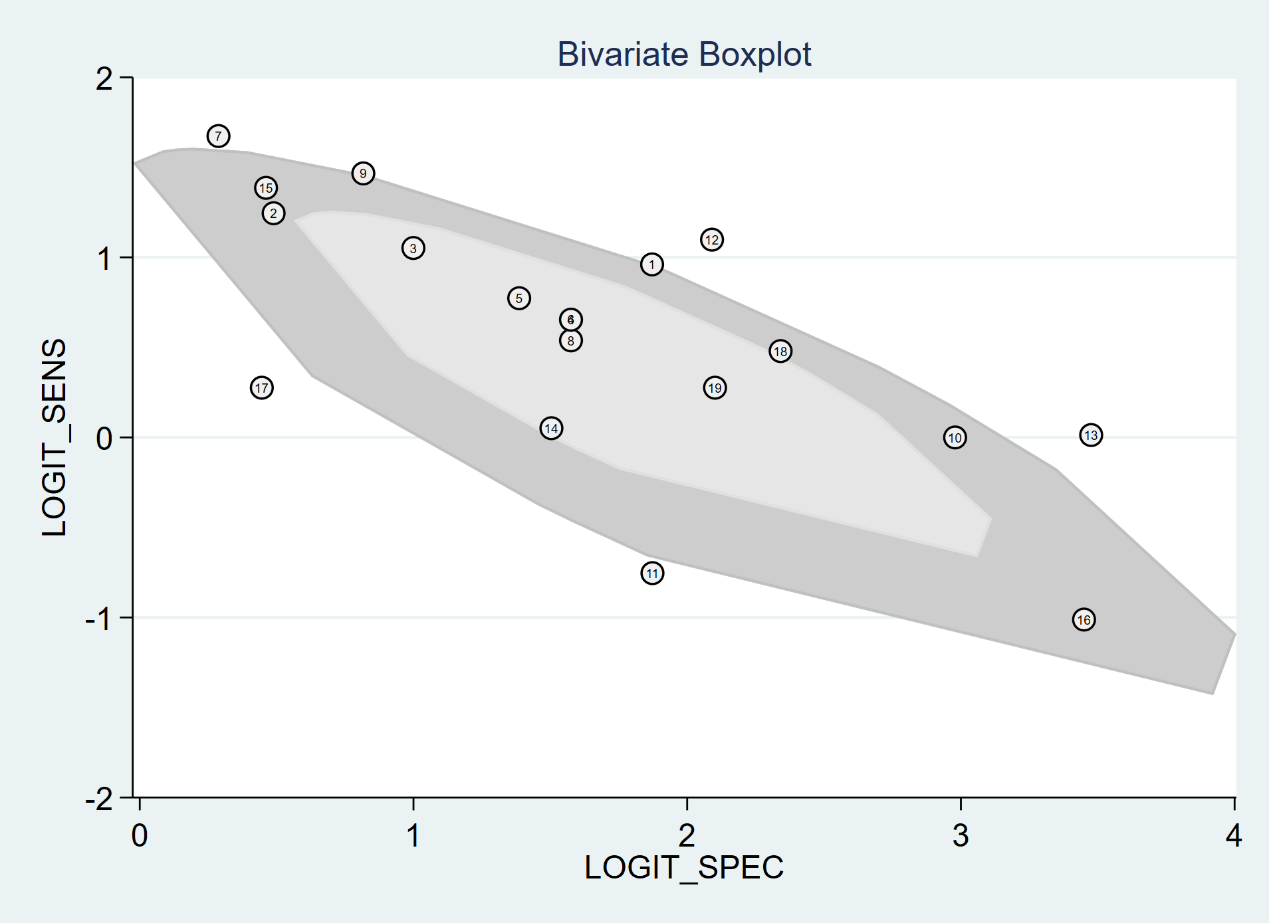
**

**C**


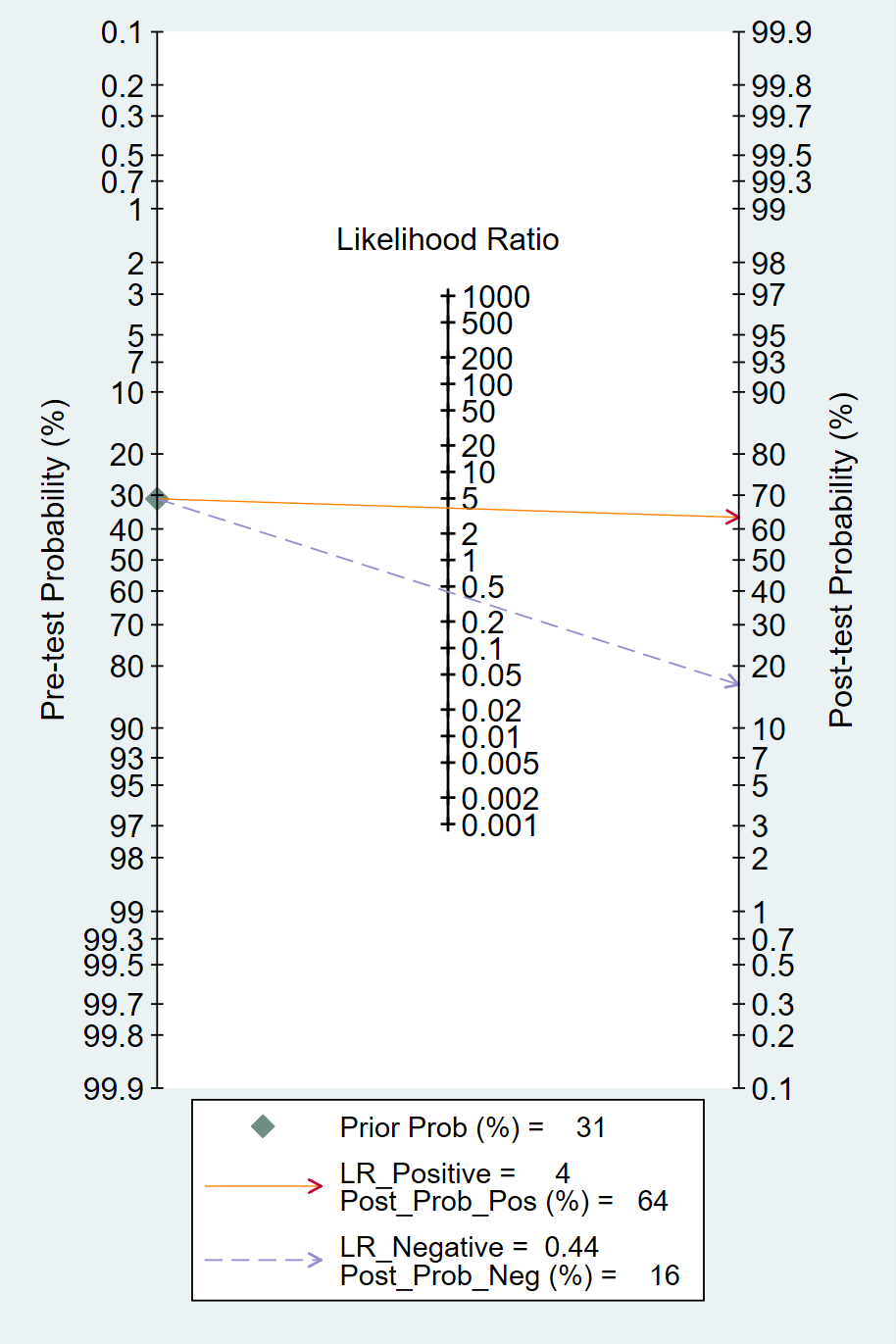


**Supplementary Figure 3.** Meta-analysis of the predictive accuracy of non-specialist clinicians with assistance of endoscopic images in diagnosis of early GC (**A**) Funnel plot for publication bias; (**B**) Heterogeneity box plot; (**C**) Clinical application nomogram

**A**

**
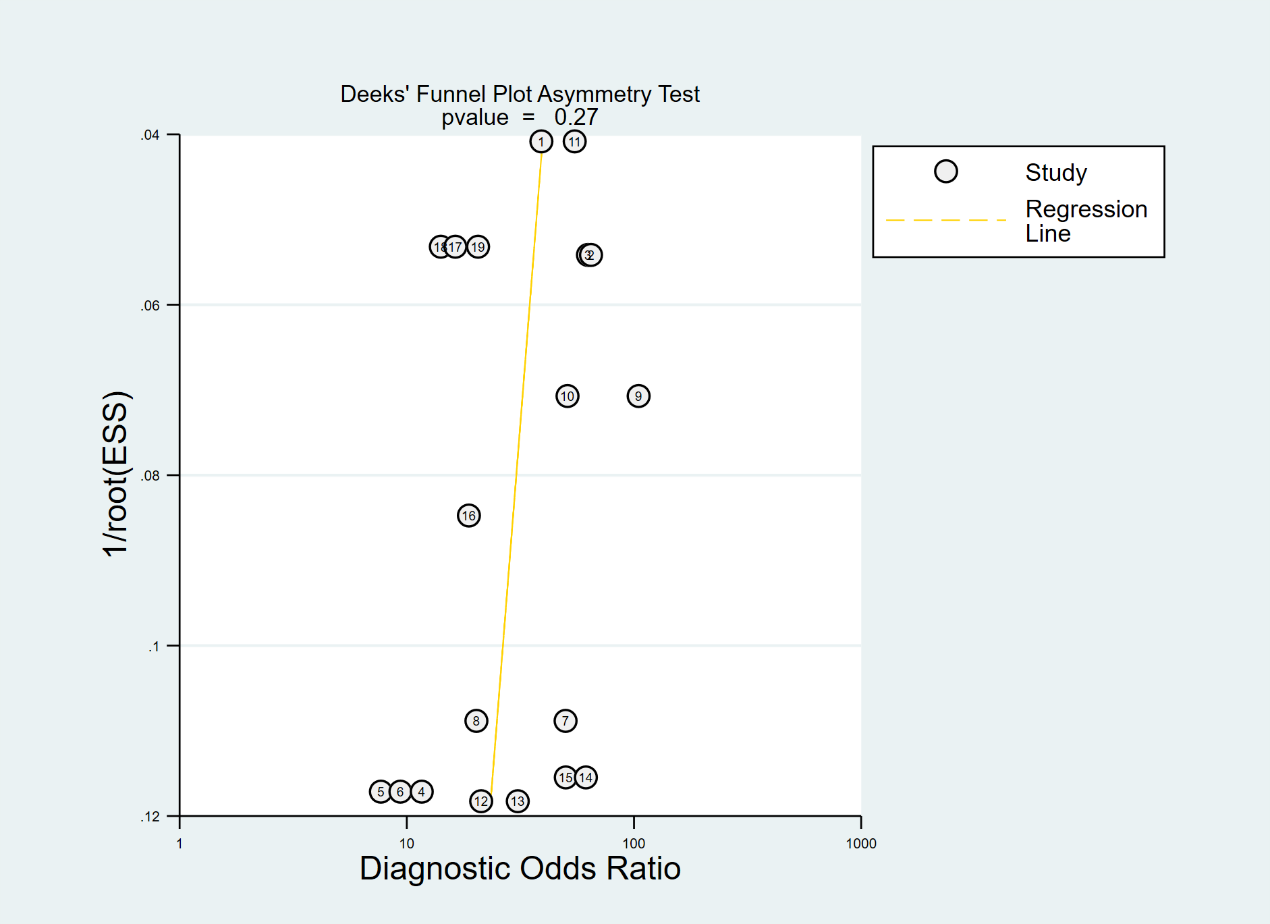
**

**B**

**
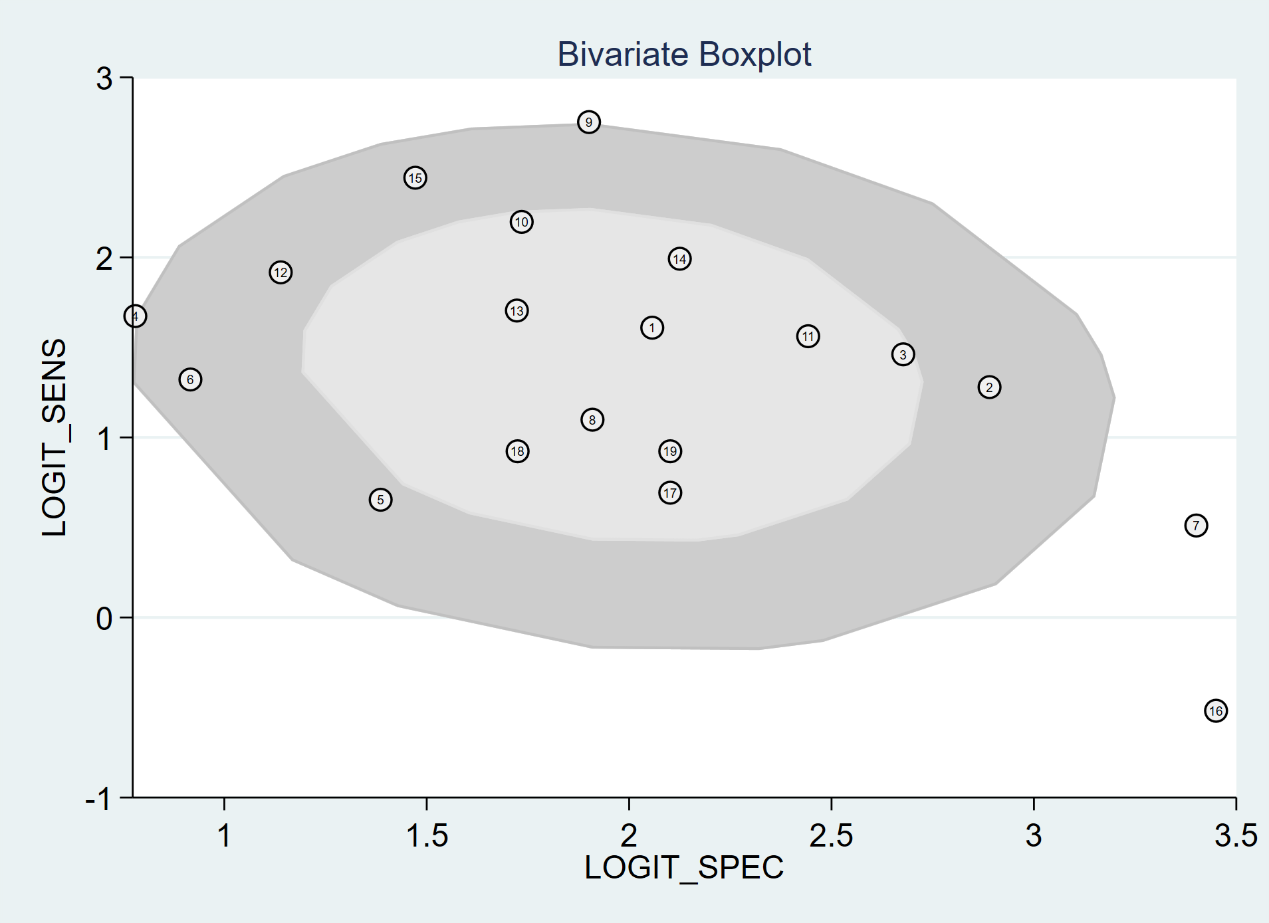
**

**C**


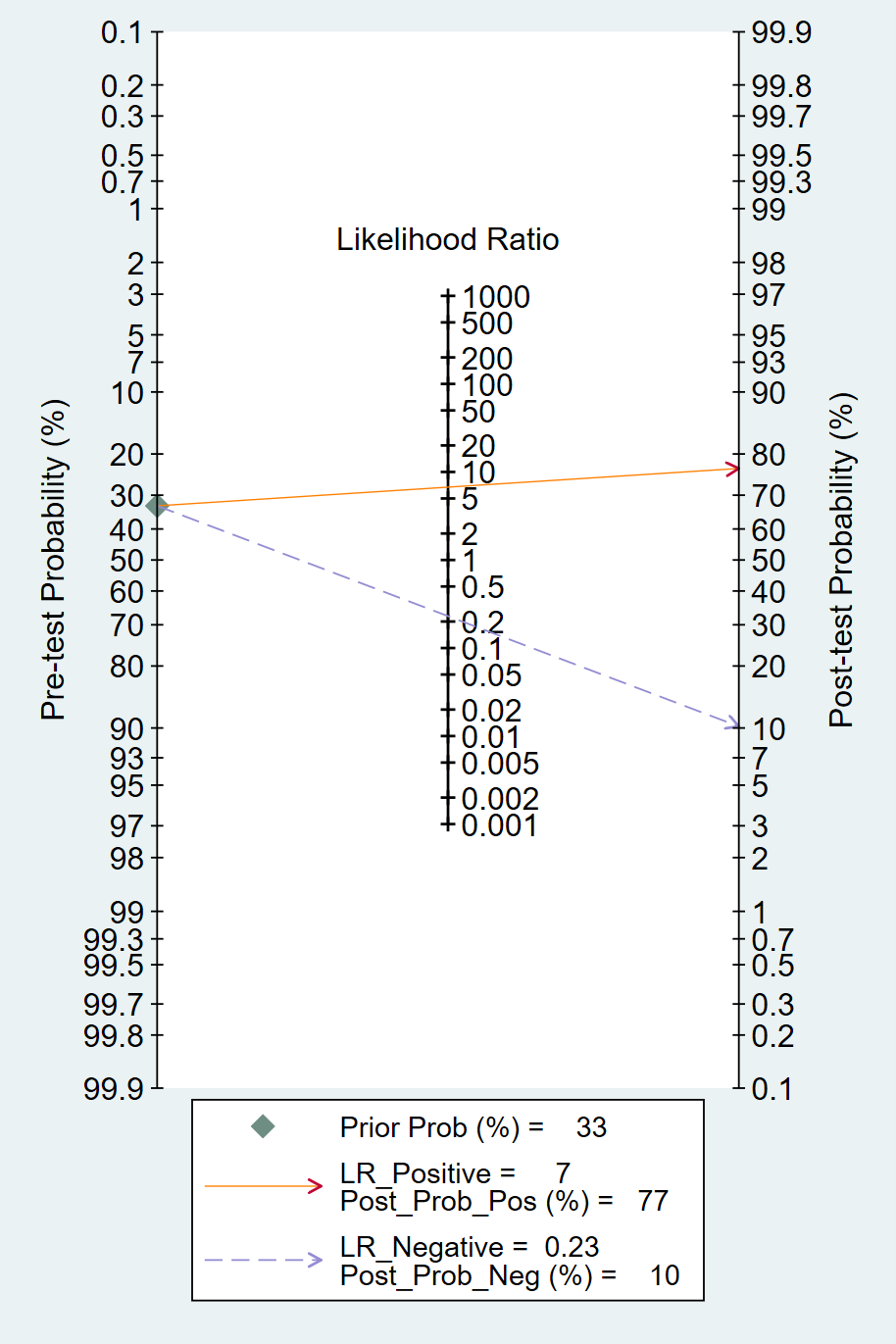


**Supplementary Figure 4.** Meta-analysis of the predictive accuracy of specialist clinicians with assistance of endoscopic images in the diagnosis of early GC (**A**) Funnel plot for publication bias; (**B**) Heterogeneity box plot; (**C**) Clinical application nomogram

**A**

**
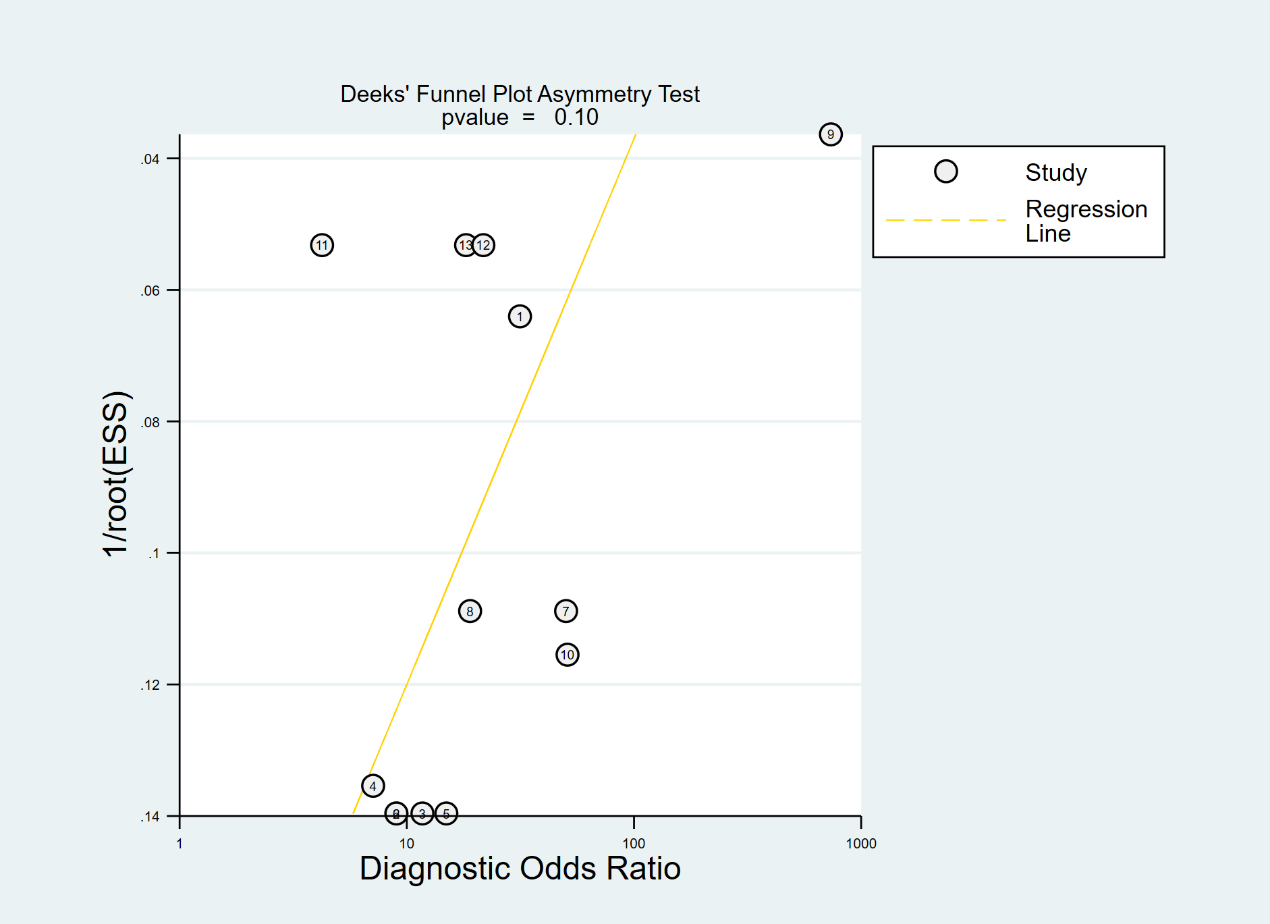
**

**B**

**
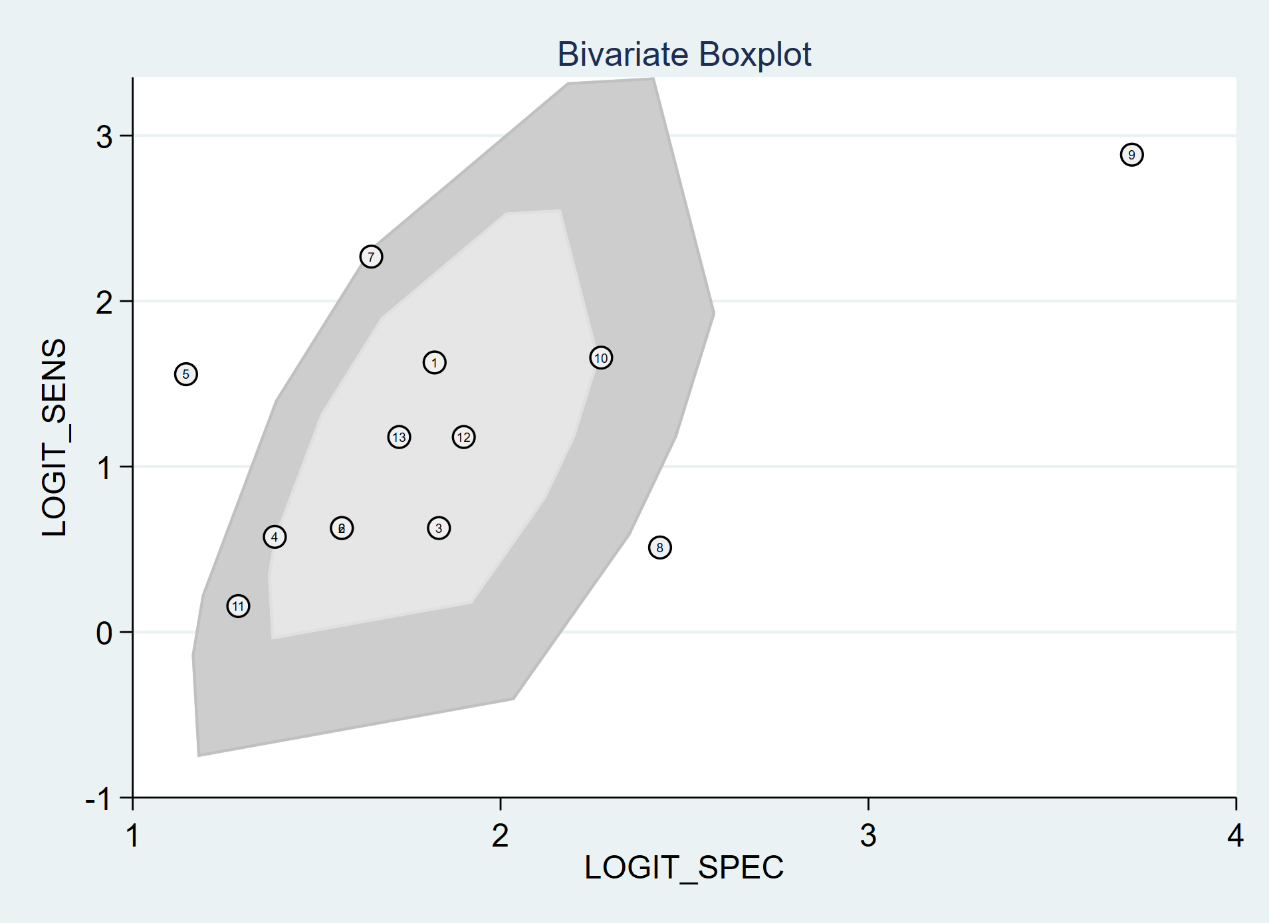
**

**C**


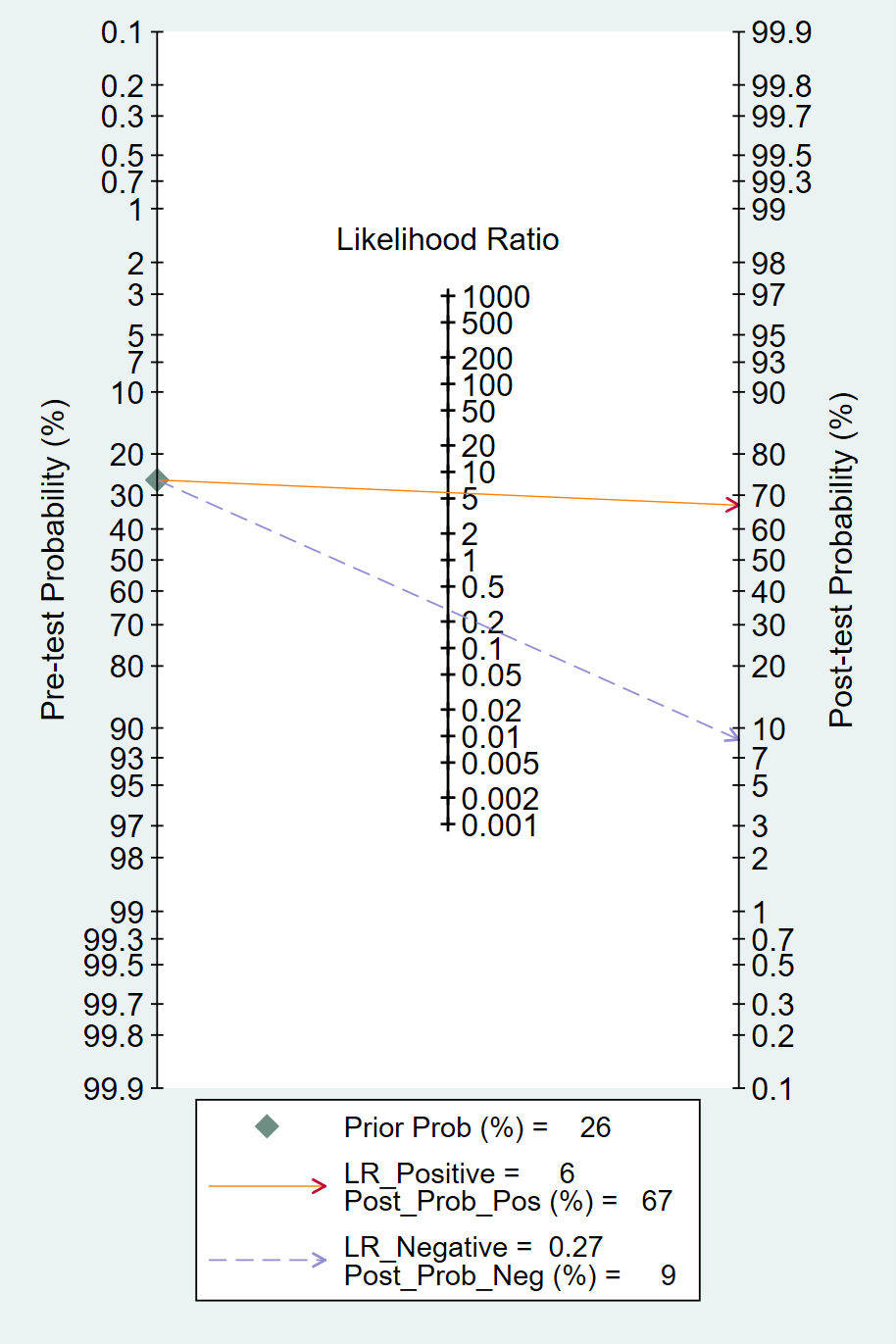


**Supplementary Figure 5.** Meta-analysis of non-specialist clinicians with assistance of the machine learning models in the diagnosis of early GC by endoscopic images (A) Funnel plot for publication bias; (B) Heterogeneity box plot; (C) Clinical application nomogram

**A**

**
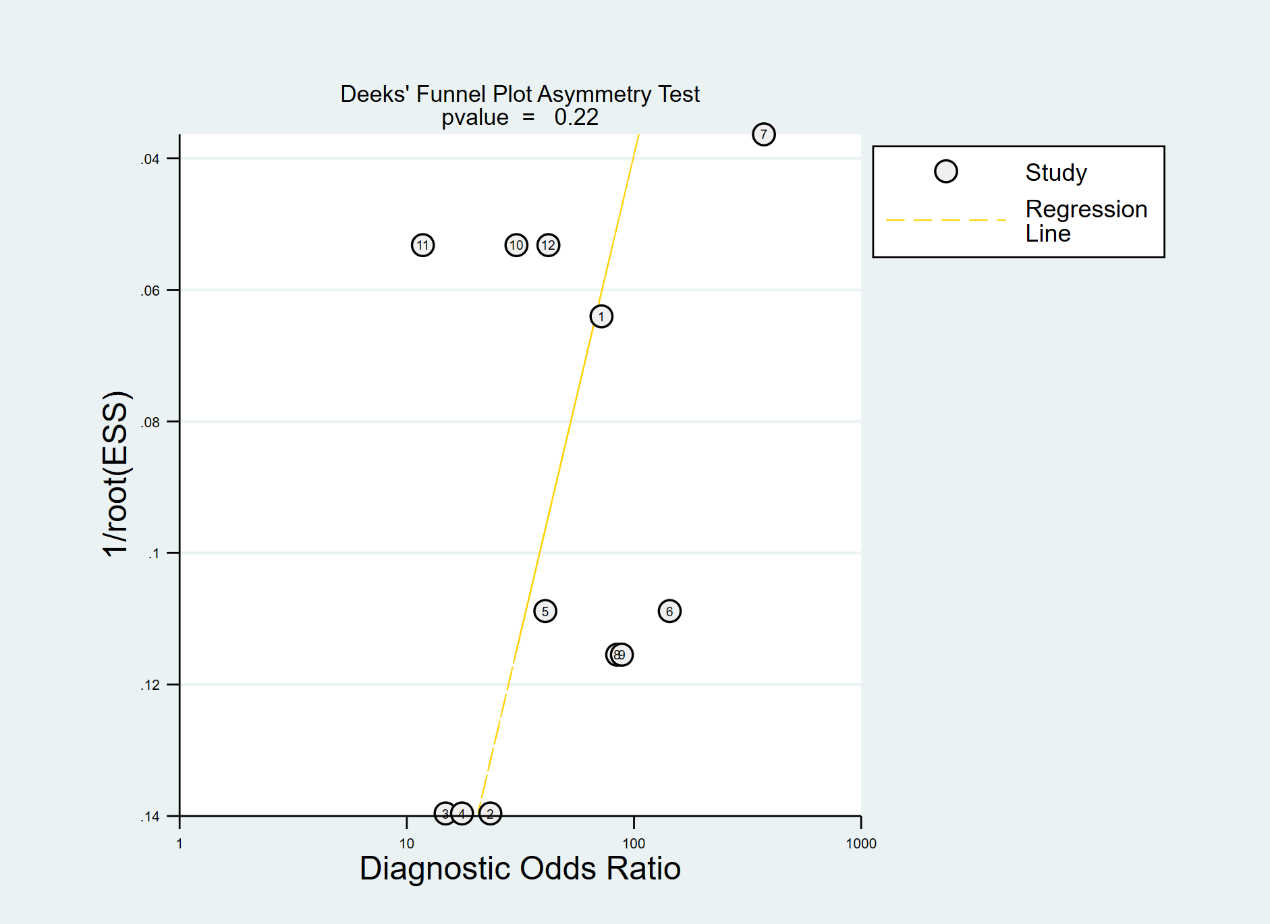
**

**B**

**
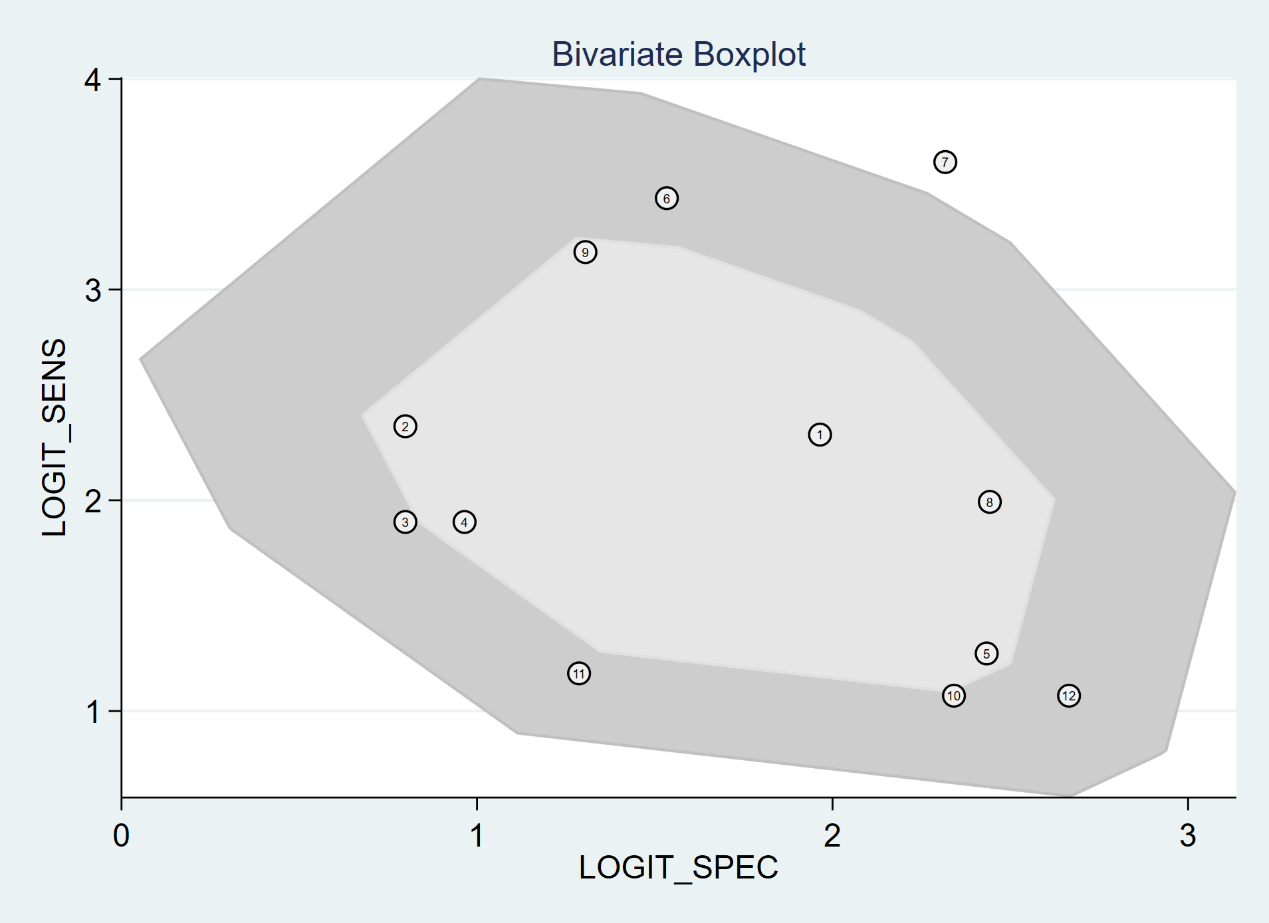
**

**C**


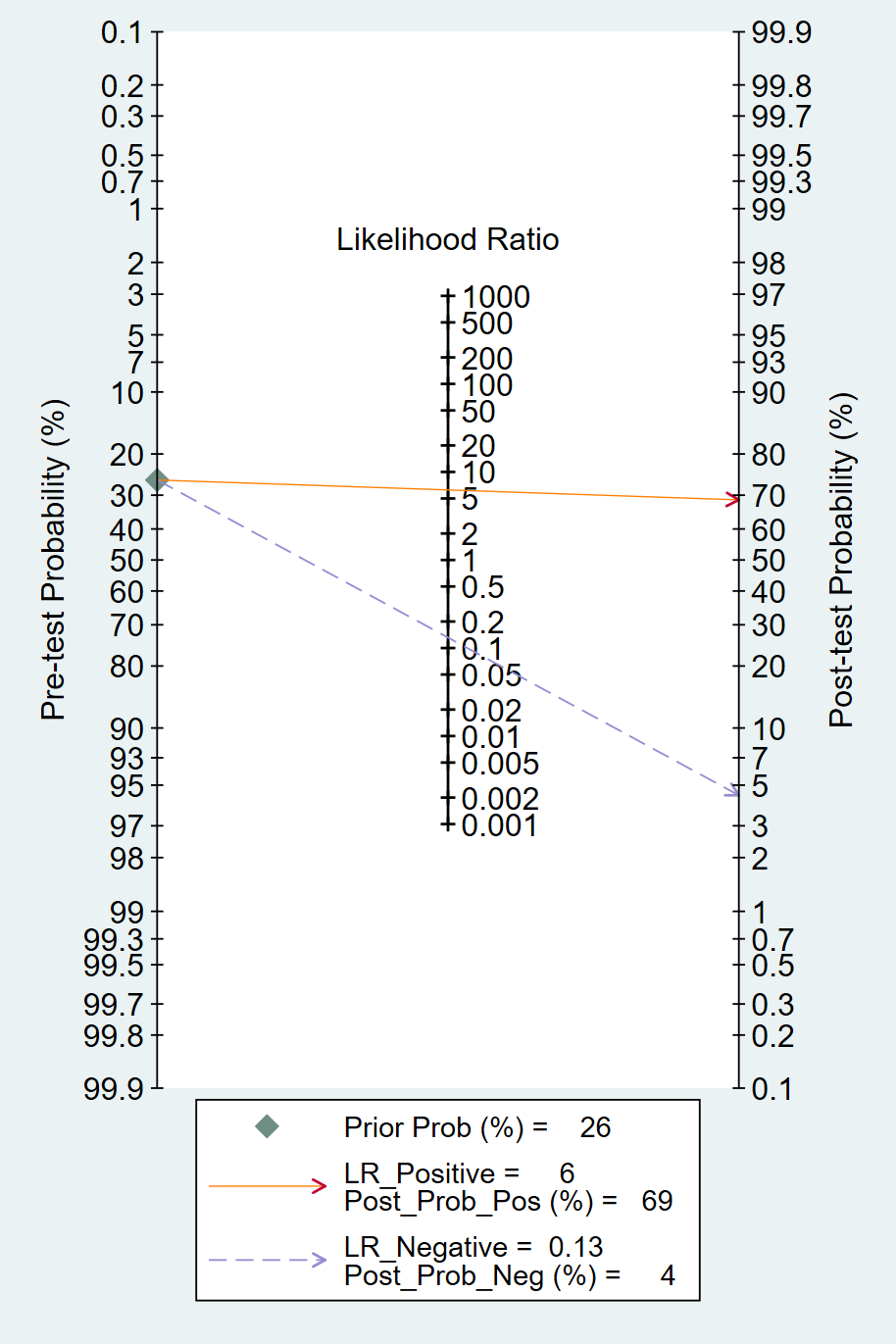


**Supplementary Figure 6.** Meta-analysis of the predictive accuracy of specialist clinicians with assistance of the machine learning models in the diagnosis of early GC by endoscopic images (**A**) Funnel plot for publication bias; (**B**) Heterogeneity box plot; (**C**) Clinical application nomogram

**A**

**
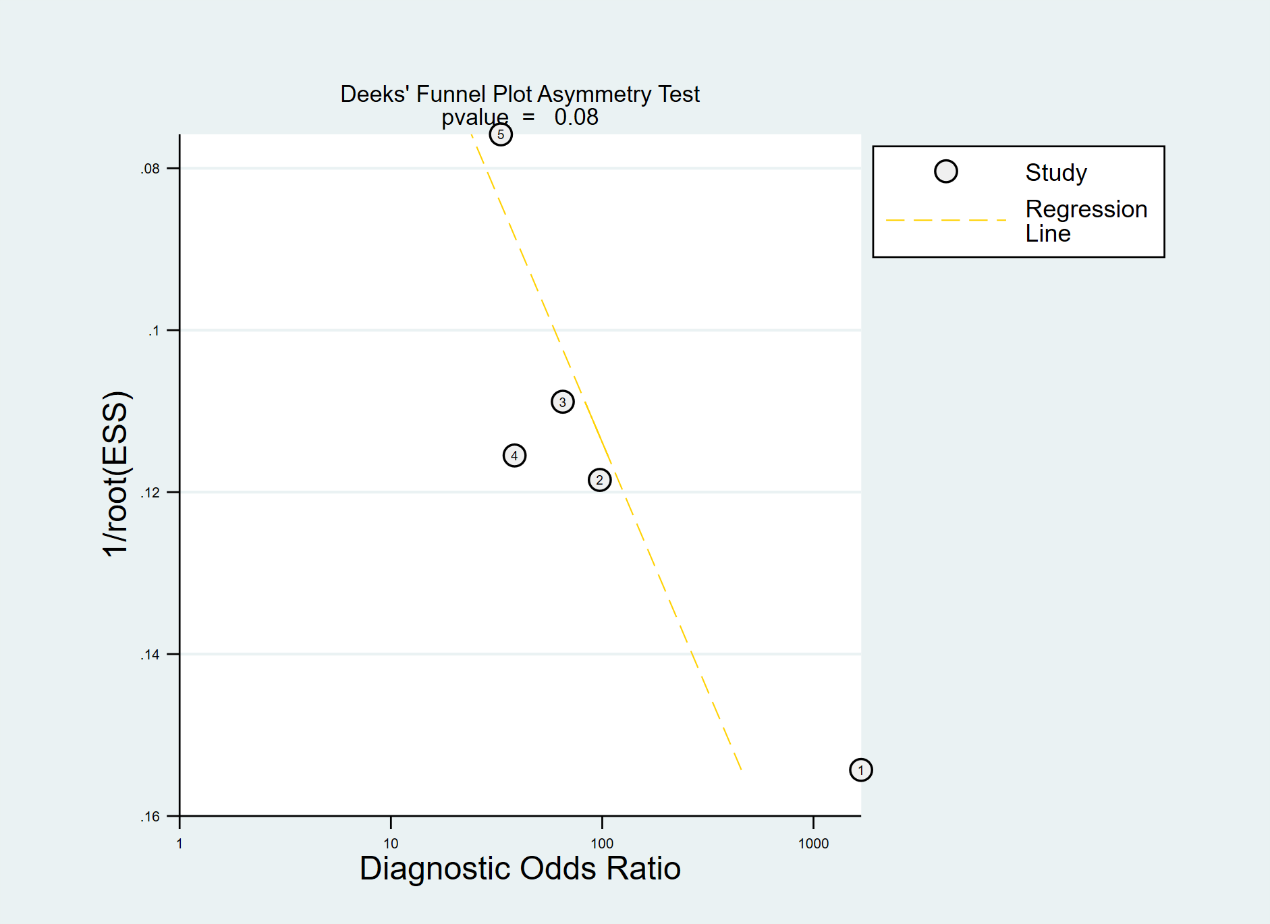
**

**B**

**
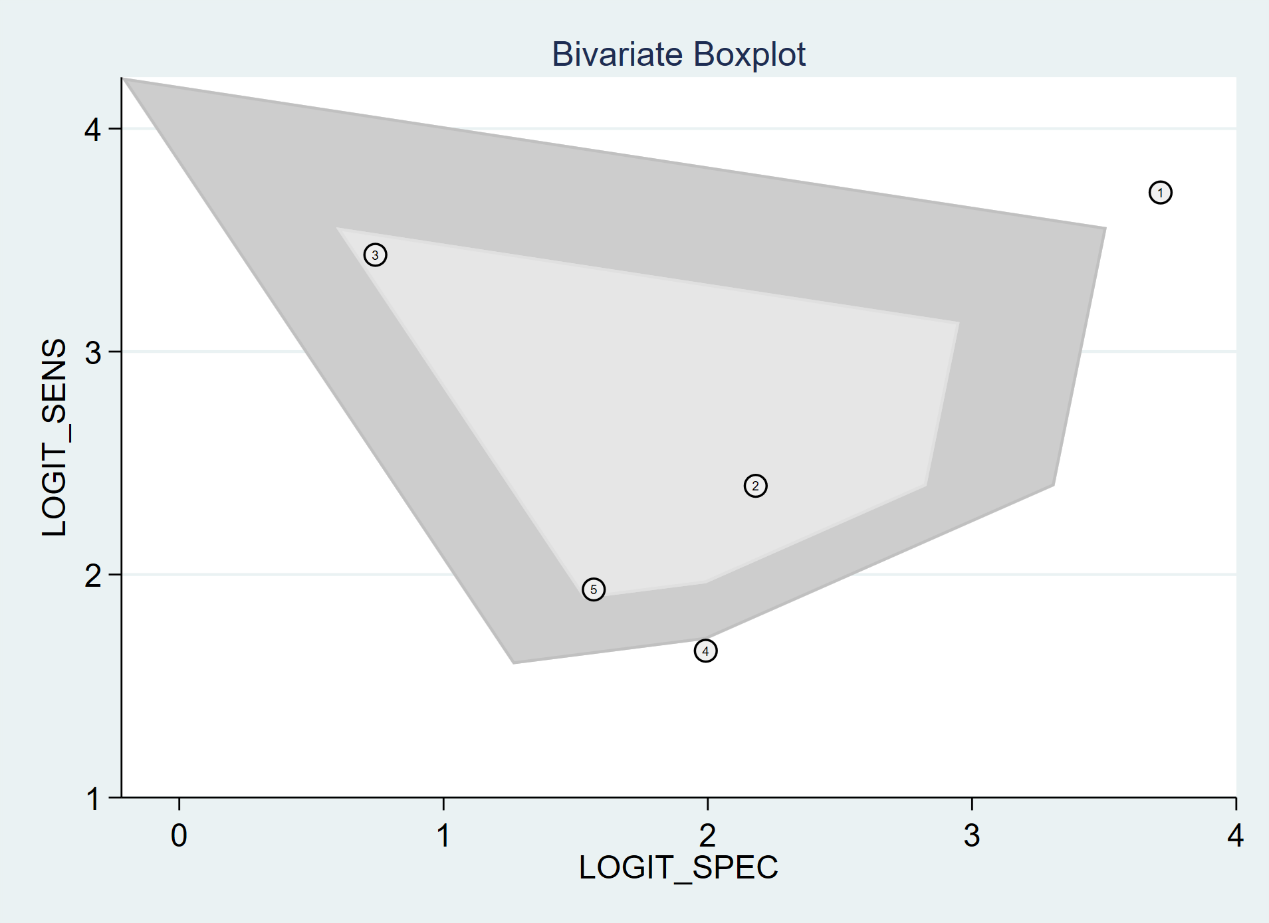
**

**C**


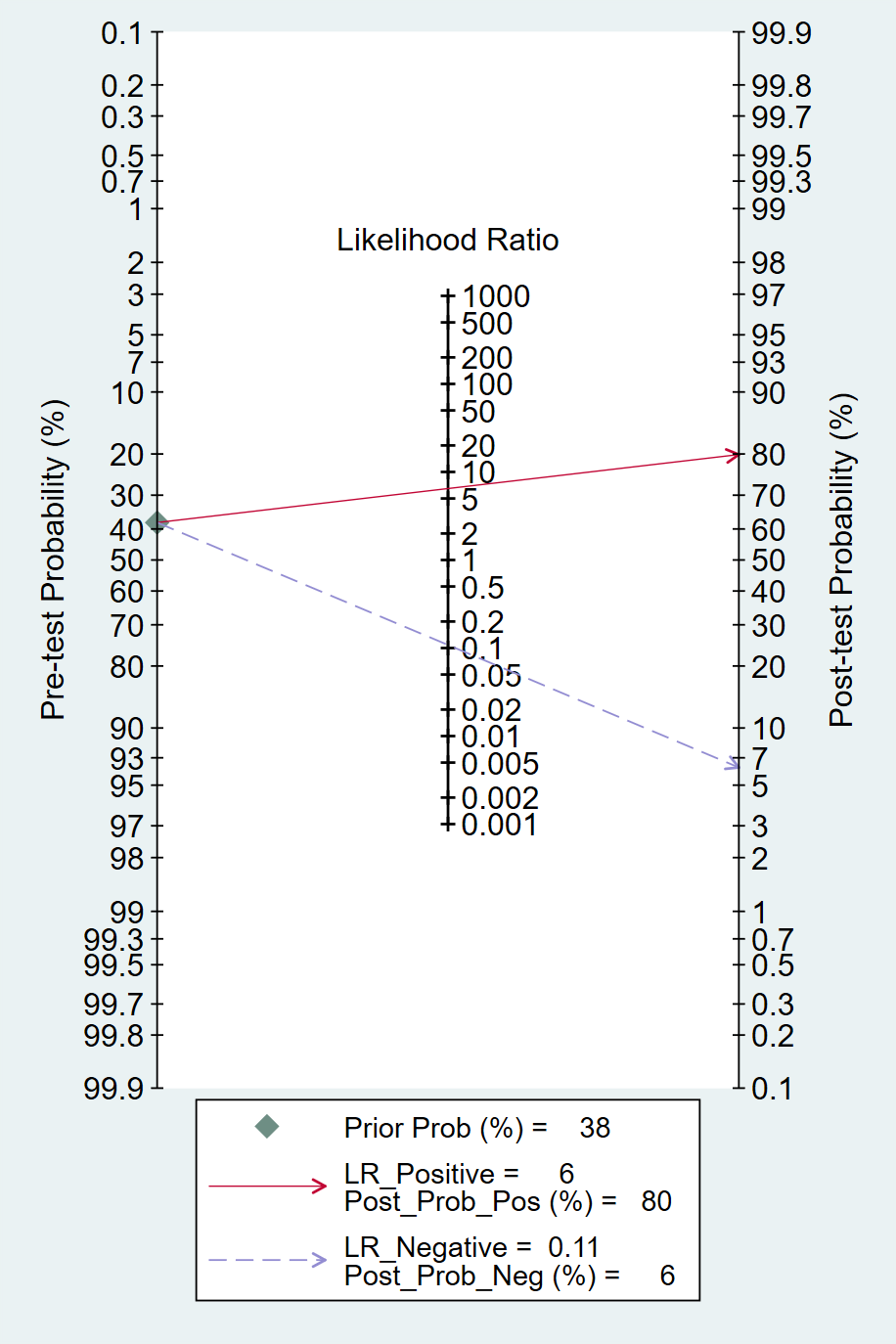


**Supplementary Figure 7.** Meta-analysis of the predictive accuracy of machine learning models in diagnosis of early GC in the video validation cohort (**A**) Funnel plot for publication bias; (**B**) Heterogeneity box plot; (**C**) Clinical application nomogram

**A**

**
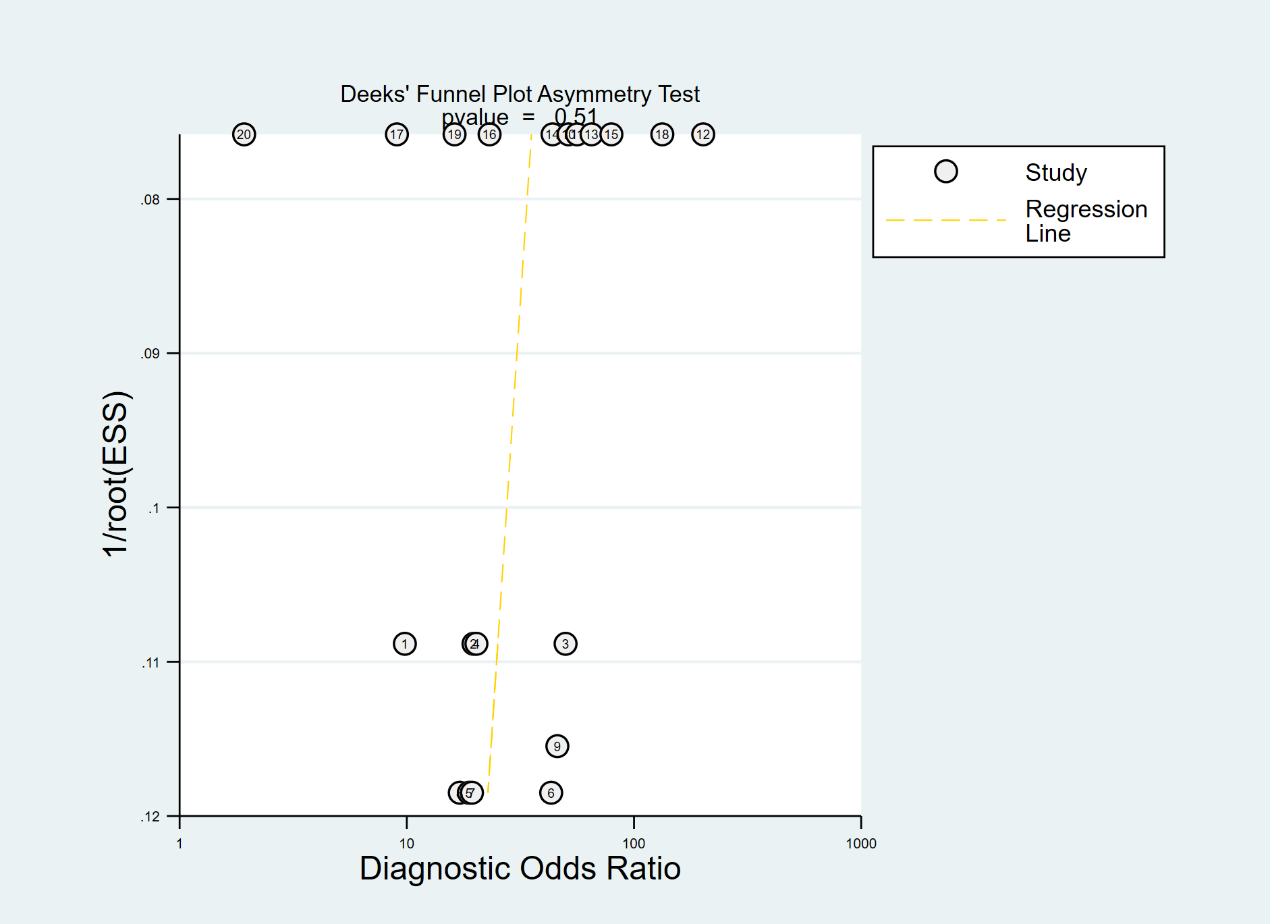
**

**B**

**
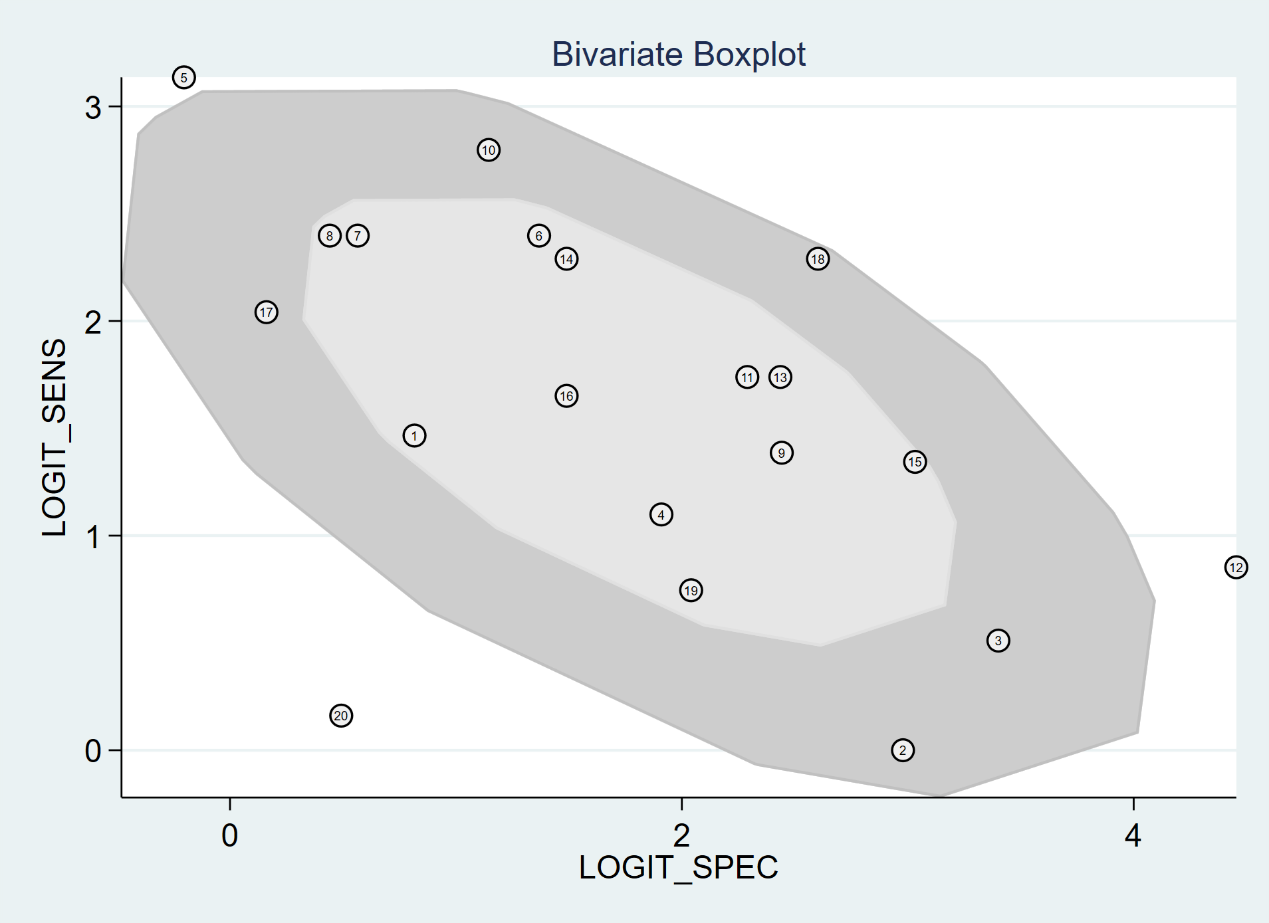
**

**C**


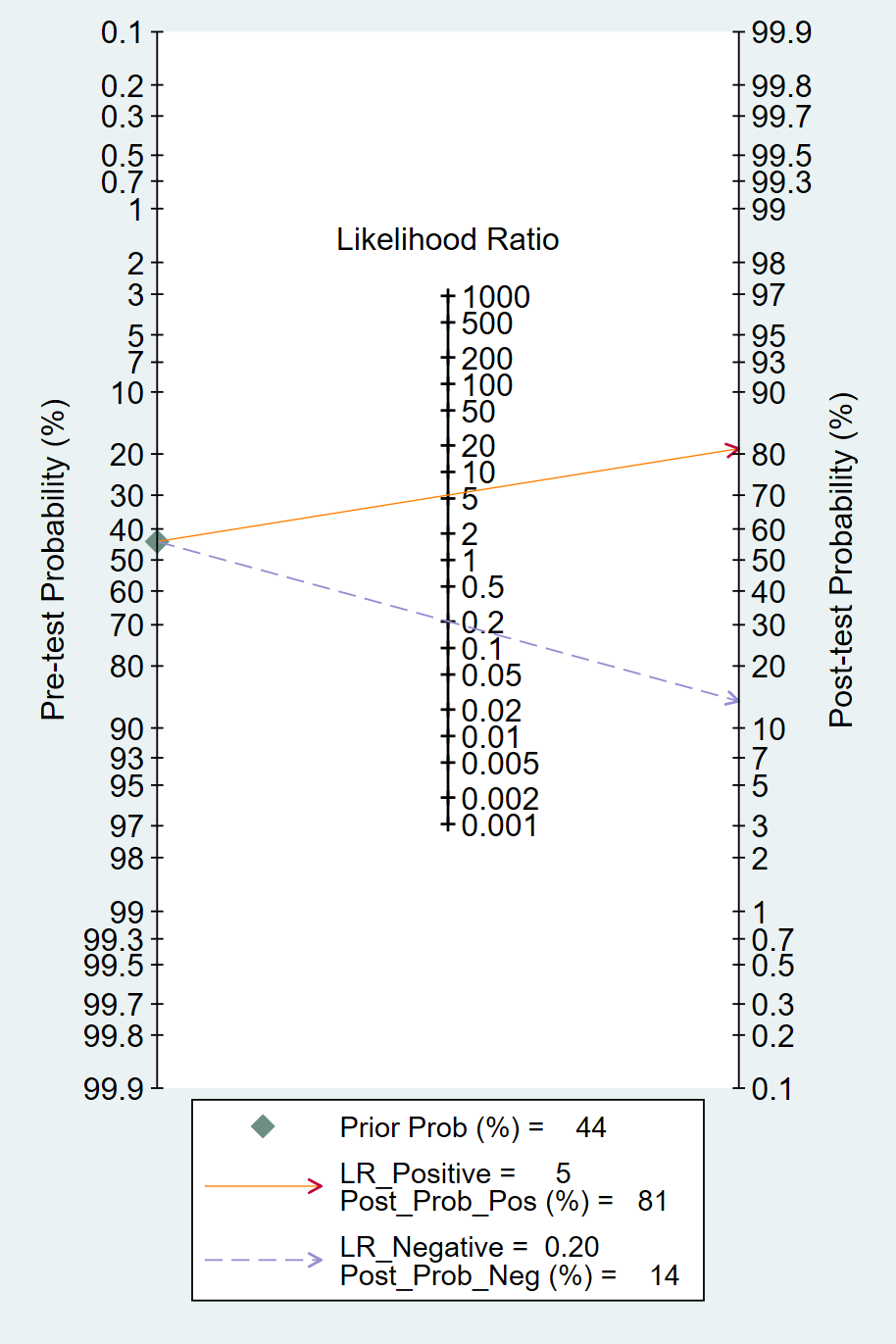


**Supplementary Figure 8.** Meta-analysis of the predictive accuracy of clinicians in diagnosis of early GC in the video validation cohort (**A**) Funnel plot for publication bias; (**B**) Heterogeneity box plot; (**C**) Clinical application nomogram
