# Supplementary material for: The value of machine learning approaches in the diagnosis of early gastric cancer: a systematic review and meta-analysis
Source: World J Surg Oncol. 2024 Feb 1;22:40. doi: 10.1186/s12957-024-03321-9 (PMC10832162; doi:10.1186/s12957-024-03321-9)
Supplement: Supplementary file 2 — Additional file 2: Table S1. Literature search strategy. Table S2. Basic characteristics of the included literature. [file 12957_2024_3321_MOESM2_ESM.docx]

# Table S1 Literature search strategy

**1.Pubmed**

| Search number | Query | Results |
| --- | --- | --- |
| #1 | "Stomach Neoplasms"[Mesh] | 107,060 |
| #2 | (((((((((((((((((((((((((((((((((((((((((((((((((((Stomach Neoplasms[Title/Abstract]) OR (Neoplasm, Stomach[Title/Abstract])) OR (Stomach Neoplasm[Title/Abstract])) OR (Neoplasms, Stomach[Title/Abstract])) OR (Gastric Neoplasms[Title/Abstract])) OR (Gastric Neoplasm[Title/Abstract])) OR (Neoplasm, Gastric[Title/Abstract])) OR (Neoplasms, Gastric[Title/Abstract])) OR (Cancer of Stomach[Title/Abstract])) OR (Stomach Cancers[Title/Abstract])) OR (Gastric Cancer[Title/Abstract])) OR (Cancer, Gastric[Title/Abstract])) OR (Cancers, Gastric[Title/Abstract])) OR (Gastric Cancers[Title/Abstract])) OR (Stomach Cancer[Title/Abstract])) OR (Cancer, Stomach[Title/Abstract])) OR (Cancers, Stomach[Title/Abstract])) OR (Cancer of the Stomach[Title/Abstract])) OR (Gastric Cancer, Familial Diffuse[Title/Abstract])) OR (stomach tumor[Title/Abstract])) OR (gastric mass (tumor[Title/Abstract]))) OR (gastric masses (tumor[Title/Abstract]))) OR (gastric neoplasia[Title/Abstract])) OR (gastric neoplasm[Title/Abstract])) OR (gastric subepithelial tumor[Title/Abstract])) OR (gastric tumor[Title/Abstract])) OR (gastric tumorigenesis[Title/Abstract])) OR (gastric tumour[Title/Abstract])) OR (mucosa tumor, stomach[Title/Abstract])) OR (mucosa tumour, stomach[Title/Abstract])) OR (neoplasia of the stomach[Title/Abstract])) OR (neoplasm of the stomach[Title/Abstract])) OR (neoplasms of the stomach[Title/Abstract])) OR (neoplastic gastric[Title/Abstract])) OR (neoplastic stomach[Title/Abstract])) OR (stomach mucosa tumor[Title/Abstract])) OR (stomach mucosa tumour[Title/Abstract])) OR (stomach neoplasia[Title/Abstract])) OR (stomach neoplasm[Title/Abstract])) OR (stomach neoplasms[Title/Abstract])) OR (stomach tumorigenesis[Title/Abstract])) OR (stomach tumour[Title/Abstract])) OR (stomach ulcerated tumor[Title/Abstract])) OR (stomach ulcerated tumour[Title/Abstract])) OR (stomach ulcerating tumor[Title/Abstract])) OR (stomach ulcerating tumour[Title/Abstract])) OR (tumor of the gastric[Title/Abstract])) OR (tumor of the stomach[Title/Abstract])) OR (tumor, stomach mucosa[Title/Abstract])) OR (tumour of the gastric[Title/Abstract])) OR (tumour of the stomach[Title/Abstract])) OR (tumour, stomach mucosa[Title/Abstract]) | 107,805 |
| #3 | ("Stomach Neoplasms"[Mesh]) OR ((((((((((((((((((((((((((((((((((((((((((((((((((((Stomach Neoplasms[Title/Abstract]) OR (Neoplasm, Stomach[Title/Abstract])) OR (Stomach Neoplasm[Title/Abstract])) OR (Neoplasms, Stomach[Title/Abstract])) OR (Gastric Neoplasms[Title/Abstract])) OR (Gastric Neoplasm[Title/Abstract])) OR (Neoplasm, Gastric[Title/Abstract])) OR (Neoplasms, Gastric[Title/Abstract])) OR (Cancer of Stomach[Title/Abstract])) OR (Stomach Cancers[Title/Abstract])) OR (Gastric Cancer[Title/Abstract])) OR (Cancer, Gastric[Title/Abstract])) OR (Cancers, Gastric[Title/Abstract])) OR (Gastric Cancers[Title/Abstract])) OR (Stomach Cancer[Title/Abstract])) OR (Cancer, Stomach[Title/Abstract])) OR (Cancers, Stomach[Title/Abstract])) OR (Cancer of the Stomach[Title/Abstract])) OR (Gastric Cancer, Familial Diffuse[Title/Abstract])) OR (stomach tumor[Title/Abstract])) OR (gastric mass (tumor[Title/Abstract]))) OR (gastric masses (tumor[Title/Abstract]))) OR (gastric neoplasia[Title/Abstract])) OR (gastric neoplasm[Title/Abstract])) OR (gastric subepithelial tumor[Title/Abstract])) OR (gastric tumor[Title/Abstract])) OR (gastric tumorigenesis[Title/Abstract])) OR (gastric tumour[Title/Abstract])) OR (mucosa tumor, stomach[Title/Abstract])) OR (mucosa tumour, stomach[Title/Abstract])) OR (neoplasia of the stomach[Title/Abstract])) OR (neoplasm of the stomach[Title/Abstract])) OR (neoplasms of the stomach[Title/Abstract])) OR (neoplastic gastric[Title/Abstract])) OR (neoplastic stomach[Title/Abstract])) OR (stomach mucosa tumor[Title/Abstract])) OR (stomach mucosa tumour[Title/Abstract])) OR (stomach neoplasia[Title/Abstract])) OR (stomach neoplasm[Title/Abstract])) OR (stomach neoplasms[Title/Abstract])) OR (stomach tumorigenesis[Title/Abstract])) OR (stomach tumour[Title/Abstract])) OR (stomach ulcerated tumor[Title/Abstract])) OR (stomach ulcerated tumour[Title/Abstract])) OR (stomach ulcerating tumor[Title/Abstract])) OR (stomach ulcerating tumour[Title/Abstract])) OR (tumor of the gastric[Title/Abstract])) OR (tumor of the stomach[Title/Abstract])) OR (tumor, stomach mucosa[Title/Abstract])) OR (tumour of the gastric[Title/Abstract])) OR (tumour of the stomach[Title/Abstract])) OR (tumour, stomach mucosa[Title/Abstract])) | 138,248 |
| #4 | "Machine Learning"[Mesh] | 48,951 |
| #5 | ((((((((((((((((((((((((machine learning[Title/Abstract]) ) OR (Transfer Learning[Title/Abstract])) OR (Deep learning[Title/Abstract])) OR (Learning, Transfer[Title/Abstract])) OR (Ensemble Learning[Title/Abstract])) OR (artificial intelligence[Title/Abstract])) OR (Prediction model[Title/Abstract])) OR (random forest[Title/Abstract])) OR (neural network[Title/Abstract])) OR (Support vector machine[Title/Abstract])) OR (SVM[Title/Abstract])) OR (Gradient Boosting Machine[Title/Abstract])) OR (GBM[Title/Abstract])) OR (Nomogram[Title/Abstract])) OR (XGboost[Title/Abstract])) OR (Adaboost[Title/Abstract])) OR (Decision tree[Title/Abstract])) OR (Risk Prediction[Title/Abstract])) OR (Risk-Prediction[Title/Abstract])) OR (Radiomics[Title/Abstract])) OR (radiomic[Title/Abstract])) OR (radiogenomic[Title/Abstract])) OR (radiomics-based[Title/Abstract])) OR (radiomic signature[Title/Abstract]) | 237,214 |
| #6 | ("Machine Learning"[Mesh]) OR (((((((((((((((((((((((((machine learning[Title/Abstract]) ) OR (Transfer Learning[Title/Abstract])) OR (Deep learning[Title/Abstract])) OR (Learning, Transfer[Title/Abstract])) OR (Ensemble Learning[Title/Abstract])) OR (artificial intelligence[Title/Abstract])) OR (Prediction model[Title/Abstract])) OR (random forest[Title/Abstract])) OR (neural network[Title/Abstract])) OR (Support vector machine[Title/Abstract])) OR (SVM[Title/Abstract])) OR (Gradient Boosting Machine[Title/Abstract])) OR (GBM[Title/Abstract])) OR (Nomogram[Title/Abstract])) OR (XGboost[Title/Abstract])) OR (Adaboost[Title/Abstract])) OR (Decision tree[Title/Abstract])) OR (Risk Prediction[Title/Abstract])) OR (Risk-Prediction[Title/Abstract])) OR (Radiomics[Title/Abstract])) OR (radiomic[Title/Abstract])) OR (radiogenomic[Title/Abstract])) OR (radiomics-based[Title/Abstract])) OR (radiomic signature[Title/Abstract])) | 242,522 |
| #7 | (("Stomach Neoplasms"[Mesh]) OR ((((((((((((((((((((((((((((((((((((((((((((((((((((Stomach Neoplasms[Title/Abstract]) OR (Neoplasm, Stomach[Title/Abstract])) OR (Stomach Neoplasm[Title/Abstract])) OR (Neoplasms, Stomach[Title/Abstract])) OR (Gastric Neoplasms[Title/Abstract])) OR (Gastric Neoplasm[Title/Abstract])) OR (Neoplasm, Gastric[Title/Abstract])) OR (Neoplasms, Gastric[Title/Abstract])) OR (Cancer of Stomach[Title/Abstract])) OR (Stomach Cancers[Title/Abstract])) OR (Gastric Cancer[Title/Abstract])) OR (Cancer, Gastric[Title/Abstract])) OR (Cancers, Gastric[Title/Abstract])) OR (Gastric Cancers[Title/Abstract])) OR (Stomach Cancer[Title/Abstract])) OR (Cancer, Stomach[Title/Abstract])) OR (Cancers, Stomach[Title/Abstract])) OR (Cancer of the Stomach[Title/Abstract])) OR (Gastric Cancer, Familial Diffuse[Title/Abstract])) OR (stomach tumor[Title/Abstract])) OR (gastric mass (tumor[Title/Abstract]))) OR (gastric masses (tumor[Title/Abstract]))) OR (gastric neoplasia[Title/Abstract])) OR (gastric neoplasm[Title/Abstract])) OR (gastric subepithelial tumor[Title/Abstract])) OR (gastric tumor[Title/Abstract])) OR (gastric tumorigenesis[Title/Abstract])) OR (gastric tumour[Title/Abstract])) OR (mucosa tumor, stomach[Title/Abstract])) OR (mucosa tumour, stomach[Title/Abstract])) OR (neoplasia of the stomach[Title/Abstract])) OR (neoplasm of the stomach[Title/Abstract])) OR (neoplasms of the stomach[Title/Abstract])) OR (neoplastic gastric[Title/Abstract])) OR (neoplastic stomach[Title/Abstract])) OR (stomach mucosa tumor[Title/Abstract])) OR (stomach mucosa tumour[Title/Abstract])) OR (stomach neoplasia[Title/Abstract])) OR (stomach neoplasm[Title/Abstract])) OR (stomach neoplasms[Title/Abstract])) OR (stomach tumorigenesis[Title/Abstract])) OR (stomach tumour[Title/Abstract])) OR (stomach ulcerated tumor[Title/Abstract])) OR (stomach ulcerated tumour[Title/Abstract])) OR (stomach ulcerating tumor[Title/Abstract])) OR (stomach ulcerating tumour[Title/Abstract])) OR (tumor of the gastric[Title/Abstract])) OR (tumor of the stomach[Title/Abstract])) OR (tumor, stomach mucosa[Title/Abstract])) OR (tumour of the gastric[Title/Abstract])) OR (tumour of the stomach[Title/Abstract])) OR (tumour, stomach mucosa[Title/Abstract]))) AND (("Machine Learning"[Mesh]) OR (((((((((((((((((((((((((machine learning[Title/Abstract]) ) OR (Transfer Learning[Title/Abstract])) OR (Deep learning[Title/Abstract])) OR (Learning, Transfer[Title/Abstract])) OR (Ensemble Learning[Title/Abstract])) OR (artificial intelligence[Title/Abstract])) OR (Prediction model[Title/Abstract])) OR (random forest[Title/Abstract])) OR (neural network[Title/Abstract])) OR (Support vector machine[Title/Abstract])) OR (SVM[Title/Abstract])) OR (Gradient Boosting Machine[Title/Abstract])) OR (GBM[Title/Abstract])) OR (Nomogram[Title/Abstract])) OR (XGboost[Title/Abstract])) OR (Adaboost[Title/Abstract])) OR (Decision tree[Title/Abstract])) OR (Risk Prediction[Title/Abstract])) OR (Risk-Prediction[Title/Abstract])) OR (Radiomics[Title/Abstract])) OR (radiomic[Title/Abstract])) OR (radiogenomic[Title/Abstract])) OR (radiomics-based[Title/Abstract])) OR (radiomic signature[Title/Abstract]))) | 1,394 |

**2.Cochrane**

| Search number | Query | Results |
| --- | --- | --- |
| #1 | MeSH descriptor: [Stomach Neoplasms] explode all trees | 2,904 |
| #2 | (Stomach Neoplasms):ti,ab,kw OR (Neoplasm, Stomach):ti,ab,kw OR (Stomach Neoplasm):ti,ab,kw OR (Neoplasms, Stomach):ti,ab,kw OR (Gastric Neoplasms):ti,ab,kw | 4,446 |
| #3 | (Gastric Neoplasm):ti,ab,kw OR (Neoplasm, Gastric):ti,ab,kw OR (Neoplasms, Gastric):ti,ab,kw OR (Cancer of Stomach):ti,ab,kw OR (Stomach Cancers):ti,ab,kw | 7,709 |
| #4 | (Gastric Cancer):ti,ab,kw OR (Cancer, Gastric):ti,ab,kw OR (Cancers, Gastric):ti,ab,kw OR (Gastric Cancers):ti,ab,kw OR (Stomach Cancer):ti,ab,kw | 9,729 |
| #5 | (Cancer, Stomach):ti,ab,kw OR (Cancers, Stomach):ti,ab,kw OR (Cancer of the Stomach):ti,ab,kw OR (Gastric Cancer, Familial Diffuse):ti,ab,kw OR (stomach tumor):ti,ab,kw | 7,162 |
| #6 | (gastric mass (tumor)):ti,ab,kw OR (gastric masses (tumor)):ti,ab,kw OR (gastric neoplasia):ti,ab,kw OR (gastric neoplasm):ti,ab,kw OR (gastric subepithelial tumor):ti,ab,kw | 1,257 |
| #7 | (gastric tumor):ti,ab,kw OR (gastric tumorigenesis):ti,ab,kw OR (gastric tumour):ti,ab,kw OR (mucosa tumor, stomach):ti,ab,kw OR (mucosa tumour, stomach):ti,ab,kw | 2,624 |
| #8 | (neoplasia of the stomach):ti,ab,kw OR (neoplasm of the stomach):ti,ab,kw OR (neoplasms of the stomach):ti,ab,kw OR (neoplastic gastric):ti,ab,kw OR (neoplastic stomach):ti,ab,kw | 3,899 |
| #9 | (stomach mucosa tumor):ti,ab,kw OR (stomach mucosa tumour):ti,ab,kw OR (stomach neoplasia):ti,ab,kw OR (stomach neoplasm):ti,ab,kw OR (stomach neoplasms):ti,ab,kw | 4,035 |
| #10 | (stomach tumorigenesis):ti,ab,kw OR (stomach tumour):ti,ab,kw OR (stomach ulcerated tumor):ti,ab,kw OR (stomach ulcerated tumour):ti,ab,kw OR (stomach ulcerating tumor):ti,ab,kw | 2,333 |
| #11 | (stomach ulcerating tumour):ti,ab,kw OR (tumor of the gastric):ti,ab,kw OR (tumor of the stomach):ti,ab,kw OR (tumor, stomach mucosa):ti,ab,kw OR (tumour of the gastric):ti,ab,kw | 3,078 |
| #12 | (tumour of the stomach):ti,ab,kw OR (tumour, stomach mucosa):ti,ab,kw | 2,271 |
| #13 | #1 or #2 or #3 or #4 or #5 or #6 or #7 or #8 or #9 or #10 or #11 or #12 | 11,103 |
| #14 | MeSH descriptor: [Machine Learning] explode all trees | 254 |
| #15 | (machine learning):ti,ab,kw OR (Transfer Learning):ti,ab,kw OR (Deep learning):ti,ab,kw OR (Learning, Transfer):ti,ab,kw OR (Ensemble Learning):ti,ab,kw | 4,260 |
| #16 | (artificial intelligence):ti,ab,kw OR (Prediction model):ti,ab,kw OR (random forest):ti,ab,kw OR (neural network):ti,ab,kw OR (Support vector machine):ti,ab,kw | 8,503 |
| #17 | (SVM):ti,ab,kw OR (Gradient Boosting Machine):ti,ab,kw OR (GBM):ti,ab,kw OR (Nomogram):ti,ab,kw OR (XGboost):ti,ab,kw | 2,505 |
| #18 | (Adaboost):ti,ab,kw OR (Decision tree):ti,ab,kw OR (Risk Prediction):ti,ab,kw OR (Risk-Prediction):ti,ab,kw OR (Radiomics):ti,ab,kw | 6,666 |
| #19 | (radiomic):ti,ab,kw OR (radiogenomic):ti,ab,kw OR (radiomics-based):ti,ab,kw OR (radiomic signature):ti,ab,kw | 254 |
| #20 | #14 or #15 or #16 or #17 or #18 or #19 | 16,849 |
| #21 | #13 and #20 | 138 |

**3.Embase**

| Search number | Query | Results |
| --- | --- | --- |
| #1 | 'stomach neoplasms'/exp OR 'stomach neoplasms' OR (('stomach'/exp OR stomach) AND ('neoplasms'/exp OR neoplasms)) | 250,342 |
| #2 | 'neoplasm, stomach':ab,ti OR 'neoplasms, stomach':ab,ti OR 'gastric neoplasms':ab,ti OR 'neoplasm, gastric':ab,ti OR 'neoplasms, gastric':ab,ti OR 'cancer of stomach':ab,ti OR 'stomach cancers':ab,ti OR 'gastric cancer':ab,ti OR 'cancer, gastric':ab,ti OR 'cancers, gastric':ab,ti OR 'gastric cancers':ab,ti OR 'stomach cancer':ab,ti OR 'cancer, stomach':ab,ti OR 'cancers, stomach':ab,ti OR 'cancer of the stomach':ab,ti OR 'gastric cancer, familial diffuse':ab,ti OR 'stomach tumor':ab,ti OR ('gastric mass':ab,ti AND tumor:ab,ti) OR ('gastric masses':ab,ti AND tumor:ab,ti) OR 'gastric neoplasia':ab,ti OR 'gastric neoplasm':ab,ti OR 'gastric subepithelial tumor':ab,ti OR 'gastric tumor':ab,ti OR 'gastric tumorigenesis':ab,ti OR 'gastric tumour':ab,ti OR 'mucosa tumor, stomach':ab,ti OR 'mucosa tumour, stomach':ab,ti OR 'neoplasia of the stomach':ab,ti OR 'neoplasm of the stomach':ab,ti OR 'neoplasms of the stomach':ab,ti OR 'neoplastic gastric':ab,ti OR 'neoplastic stomach':ab,ti OR 'stomach mucosa tumor':ab,ti OR 'stomach mucosa tumour':ab,ti OR 'stomach neoplasia':ab,ti OR 'stomach neoplasm':ab,ti OR 'stomach neoplasms':ab,ti OR 'stomach tumorigenesis':ab,ti OR 'stomach tumour':ab,ti OR 'stomach ulcerated tumor':ab,ti OR 'stomach ulcerated tumour':ab,ti OR 'stomach ulcerating tumor':ab,ti OR 'stomach ulcerating tumour':ab,ti OR 'tumor of the gastric':ab,ti OR 'tumor of the stomach':ab,ti OR 'tumor, stomach mucosa':ab,ti OR 'tumour of the gastric':ab,ti OR 'tumour of the stomach':ab,ti OR 'tumour, stomach mucosa':ab,ti | 117,084 |
| #3 | #1 OR #2 | 260,658 |
| #4 | 'machine learning':ab,ti OR 'transfer learning':ab,ti OR 'deep learning':ab,ti OR 'learning, transfer':ab,ti OR 'ensemble learning':ab,ti OR 'artificial intelligence':ab,ti OR 'prediction model':ab,ti OR 'random forest':ab,ti OR 'neural network':ab,ti OR 'support vector machine':ab,ti OR svm:ab,ti OR 'gradient boosting machine':ab,ti OR gbm:ab,ti OR nomogram:ab,ti OR xgboost:ab,ti OR adaboost:ab,ti OR 'decision tree':ab,ti OR 'risk prediction':ab,ti OR 'risk prediction':ab,ti OR radiomics:ab,ti OR radiomic:ab,ti OR radiogenomic:ab,ti OR 'radiomics based':ab,ti OR 'radiomic signature':ab,ti | 350,543 |
| #5 | 'machine learning'/exp | 333,710 |
| #6 | #4 OR #5 | 539,184 |
| #7 | #3 AND #6 | 3,866 |

**4.Web of science**

| Search number | Query | Results |
| --- | --- | --- |
| #1 | Stomach Neoplasms (Topic) OR Neoplasm, Stomach (Topic) OR Stomach Neoplasm (Topic) OR Neoplasms, Stomach (Topic) OR Gastric Neoplasms (Topic) OR Gastric Neoplasm (Topic) OR Neoplasm, Gastric (Topic) OR Neoplasms, Gastric (Topic) OR Cancer of Stomach (Topic) OR Stomach Cancers (Topic) OR Gastric Cancer (Topic) OR Cancer, Gastric (Topic) OR Cancers, Gastric (Topic) OR Gastric Cancers (Topic) OR Stomach Cancer (Topic) OR Cancer, Stomach (Topic) OR Cancers, Stomach (Topic) OR Cancer of the Stomach (Topic) OR Gastric Cancer, Familial Diffuse (Topic) OR stomach tumor (Topic) OR gastric mass (tumor) (Topic) OR gastric masses (tumor) (Topic) OR gastric neoplasia (Topic) OR gastric neoplasm (Topic) OR gastric subepithelial tumor (Topic) OR gastric tumor (Topic) OR gastric tumorigenesis (Topic) OR gastric tumour (Topic) OR mucosa tumor, stomach (Topic) OR mucosa tumour, stomach (Topic) OR neoplasia of the stomach (Topic) OR neoplasm of the stomach (Topic) OR neoplasms of the stomach (Topic) OR neoplastic gastric (Topic) OR neoplastic stomach (Topic) OR stomach mucosa tumor (Topic) OR stomach mucosa tumour (Topic) OR stomach neoplasia (Topic) OR stomach neoplasm (Topic) OR stomach neoplasms (Topic) OR stomach tumorigenesis (Topic) OR stomach tumour (Topic) OR stomach ulcerated tumor (Topic) OR stomach ulcerated tumour (Topic) OR stomach ulcerating tumor (Topic) OR stomach ulcerating tumour (Topic) OR tumor of the gastric (Topic) OR tumor of the stomach (Topic) OR tumor, stomach mucosa (Topic) OR tumour of the gastric (Topic) OR tumour of the stomach (Topic) OR tumour, stomach mucosa (Topic) | 148,690 |
| #2 | machine learning (Topic) OR Transfer Learning (Topic) OR Deep learning (Topic) OR Learning, Transfer (Topic) OR Ensemble Learning (Topic) OR artificial intelligence (Topic) OR Prediction model (Topic) OR random forest (Topic) OR neural network (Topic) OR Support vector machine (Topic) OR SVM (Topic) OR Gradient Boosting Machine (Topic) OR GBM (Topic) OR Nomogram (Topic) OR XGboost (Topic) OR Adaboost (Topic) OR Decision tree (Topic) OR Risk Prediction (Topic) OR Risk-Prediction (Topic) OR Radiomics (Topic) OR radiomic (Topic) OR radiogenomic (Topic) OR radiomics-based (Topic) OR radiomic signature (Topic) | 1,949,018 |
| #3 | #2 AND #1 | 3,360 |

**Table S2** Basic characteristics of the included literature

| **First author** | **Year of publication** | **Author country** | **Source of patients** | **Number of EGC cases** | **Total number of cases** | **Number of EGC images** | **Total number of images** | **Number of EGC cases in training set** | **Total number of cases in training set** | **Total number of images in training set** | **Number of EGC cases in validation set** | **Total number of cases in validation set** | **Total number of images in validation set** | **Model type** | **Modeling variables** | **Clinician comparison** |
| --- | --- | --- | --- | --- | --- | --- | --- | --- | --- | --- | --- | --- | --- | --- | --- | --- |
| Yao, Z. | 2022 | China | Multicenter | 317 | 1995 | 1384 | 47978 | 222 | 1653 | 43145 | 95 | 342 | 4833 | CNN(DarkNet53) | Image (WLI) | NO |
| Ueyama, H. | 2021 | Japan | Single center | 349 | 745 | 3797 | 5574 | 267 | 267 | 4460 | 82 | 82 | 3414 | CNN(ResNet50) | Image (ME-NBI) | NO |
| Tang, D. | 2022 | China | Multicenter | 1093 | 1260 | 18554 | 21785 | 810 | 810 | 13151 | 450 | 450 | 8634 | DCNN | Image (NBI) video | YES 3seniors 4juniors |
| Miyaki, R. | 2015 | Japan | Single center | 100 | 140 | 687 | 1330 | NA | NA | 1090 | NA | NA | 240 | SVM | Image (BLI) | NO |
| Li,Y. | 2019 | China | Single center | 29 | 76 | 2900 | 6100 | NA | NA | 4575 | NA | NA | 1525 | PLS-DA SVM | Image (Fluorescence  spectral) | NO |
| Li, L. | 2020 | China | Multicenter | 170 | 341 | 1702 | 2088 | 170 | 341 | 20000 | 170 | 341 | 341 | CNN(Inception-v3) | Image (M-NBI) | YES 2exports 2non-exports |
| Jin, T. | 2022 | China | Multicenter | NA | NA | 2237 | 227279 | NA | NA | 220918 | NA | NA | 6361 | CNN(YOLO-v4) | Image (WLI) | NO |
| Hu, H. | 2021 | China | Multicenter | 128 | 295 | NA | 1777 | 74 | 170 | NA | 56 | 125 | NA | CNN(VGG-19) | Image (ME-NBI) | YES 3seniors 5juniors |
| He, X. | 2021 | China | Multicenter | 1042 | 3099 | 1950 | 4667 | 832 | 1446 | 3734 | 210 | 365 | 933 | CNN(ResNet-50) | Image (M-IEE) video | YES 6seniors 2juniors |
| Zhou, B. | 2022 | China | Single center | 194 | 194 | 2182 | 5770 | 130 | 130 | 4527 | 64 | 64 | 1243 | CNN(EfficientDet) | Image (WLI) | NO |
| Zhang, L. M. | 2021 | China | Single center | 328 | 1358 | 6389 | 22308 | 286 | 1121 | 21217 | 42 | 237 | 1091 | CNN (ResNet34, DeepLabv3) | Image (WLI) | YES 10endoscopists |
| Wu, L. | 2019 | China | Single center | 2864 | 9351 | NA | 24549 | 2204 | 7321 | 19503 | 660 | 2030 | 5046 | DCNN (VGG-16, ResNet-50) | Image (NBI, BLI, WLI) video | YES 8seniors 6exports 7novices |
| Wu, L. | 2021 | China | Multicenter | NA | 1050 | NA | 30 videos | NA | NA | 2524 30 video | NA | 819 | NA | CNN (VGG-16, ResNet-50) | Image (WLI､MIEE) video | NO |
| Tang, D. | 2020 | China | Multicenter | NA | 1424 | 30325 | 45240 | NA | 1085 | 35823 | NA | 279 | 10931 | DCNN (Darknet-53) | Image (WLI) video | YES 6Exports 10Trainees |
| Noda, H. | 2022 | Japan | Single center | 130 | 198 | 1219 | 2171 | 61 | 126 | 1623 | 39 | 72 | 548 | CNN (ResNet50) | Image (ECS) | YES 2seniors 1juniors |
| Li, J. | 2022 | China | Single center | 363 | 692 | 679 | 1897 | NA | 567 | 1630 | NA | 125 | 267 | CNN(ResNet-50) | Image (M-IEE) video | YES 2Exports 2seniors 4novices |
| Kanesaka, T. | 2018 | Japan | Single center | 127 | 147 | 127 | 207 | NA | NA | 126 | NA | NA | 81 | SVM | Image (M-NBI) | NO |
| Ikenoyama, Y. | 2021 | Japan | Single center | NA | 2779 | 10683 | 16524 | NA | 2639 | 13584 | 75 | 140 | 2940 | CNN (SSD) | Image (WLI) | YES 33exports 34non-exports |
| Horiuchi, Y. | 2020 | Japan | Single center | 87 | 395 | 1492 | 2570 | NA | NA | 2570 | NA | 82 | 174 | CNN (GoogLeNet) | Image (ME-NBI) video | YES 11exports |
| Horiuchi, Y. | 2020 | Japan | Single center | NA | NA | 1643 | 2828 | NA | NA | 2570 | NA | NA | 258 | CNN (GoogLeNet) | Image (ME-NBI) | NO |
| Gong, L. | 2022 | China | Multicenter | NA | 294 | NA | 1886 | NA | 144 | 994 | NA | 294 | 892 | CNN (ResNet101) | Image (ME-NBI) | NO |
